# Supplementary material for: STAREG: Statistical replicability analysis of high throughput experiments with applications to spatial transcriptomic studies
Source: PLoS Genet. 2024 Oct 3;20(10):e1011423. doi: 10.1371/journal.pgen.1011423 (PMC11478871; doi:10.1371/journal.pgen.1011423)
Supplement: S1 Appendix — Including the detailed derivations of STAREG and competing methods, additional simulation studies, and data analysis results. (PDF) [file pgen.1011423.s004.pdf]

# A Estimating the unknowns with the EM algorithm

In this section, we show how to estimate unknown parameters and functions involved in $\text{Lfdr}_i$ from available paired $p$-value sequences: $(p_{1i}, p_{2i}), i = 1, \ldots, m$. Based on the four-group model, the posterior probability mass function $P(\theta_{1i}, \theta_{2i} | p_{1i}, p_{2i})$ can be written as

$$\begin{aligned}
\gamma_{i,00} &= P(\theta_{1i} = 0, \theta_{2i} = 0 \mid p_{1i}, p_{2i}) \\
&= \frac{\xi_{00} f_0(p_{1i}) f_0(p_{2i})}{\xi_{00} f_0(p_{1i}) f_0(p_{2i}) + \xi_{01} f_0(p_{1i}) f_2(p_{2i}) + \xi_{10} f_1(p_{1i}) f_0(p_{2i}) + \xi_{11} f_1(p_{1i}) f_2(p_{2i})}, \\
\gamma_{i,01} &= P(\theta_{1i} = 0, \theta_{2i} = 1 \mid p_{1i}, p_{2i}) \\
&= \frac{\xi_{01} f_0(p_{1i}) f_2(p_{2i})}{\xi_{00} f_0(p_{1i}) f_0(p_{2i}) + \xi_{01} f_0(p_{1i}) f_2(p_{2i}) + \xi_{10} f_1(p_{1i}) f_0(p_{2i}) + \xi_{11} f_1(p_{1i}) f_2(p_{2i})}, \\
\gamma_{i,10} &= P(\theta_{1i} = 1, \theta_{2i} = 0 \mid p_{1i}, p_{2i}) \\
&= \frac{\xi_{10} f_1(p_{1i}) f_0(p_{2i})}{\xi_{00} f_0(p_{1i}) f_0(p_{2i}) + \xi_{01} f_0(p_{1i}) f_2(p_{2i}) + \xi_{10} f_1(p_{1i}) f_0(p_{2i}) + \xi_{11} f_1(p_{1i}) f_2(p_{2i})}, \\
\gamma_{i,11} &= P(\theta_{1i} = 1, \theta_{2i} = 1 \mid p_{1i}, p_{2i}) \\
&= \frac{\xi_{11} f_1(p_{1i}) f_2(p_{2i})}{\xi_{00} f_0(p_{1i}) f_0(p_{2i}) + \xi_{01} f_0(p_{1i}) f_2(p_{2i}) + \xi_{10} f_1(p_{1i}) f_0(p_{2i}) + \xi_{11} f_1(p_{1i}) f_2(p_{2i})},
\end{aligned}$$

where $f_0$ is the density function of standard uniform distribution. We need to estimate unknown parameters $\xi_{00}, \xi_{01}, \xi_{10}, \xi_{11}$ and unknown functions $f_1, f_2$. With a reasonable initialization of the unknowns $(\hat{\xi}_{00}^{(0)}, \hat{\xi}_{01}^{(0)}, \hat{\xi}_{10}^{(0)}, \hat{\xi}_{11}^{(0)}, \hat{f}_1^{(0)}, \hat{f}_2^{(0)})$, we can derive an EM algorithm [1] by iteratively implementing the following two steps.

**E-step:** Given current estimates of $(\hat{\xi}_{00}^{(t)}, \hat{\xi}_{01}^{(t)}, \hat{\xi}_{10}^{(t)}, \hat{\xi}_{11}^{(t)}, \hat{f}_1^{(t)}, \hat{f}_2^{(t)})$, calculate $\gamma_{i,00}^{(t)}, \gamma_{i,01}^{(t)}, \gamma_{i,10}^{(t)}, \gamma_{i,11}^{(t)}$,

respectively. Define conditional expectation of the log-likelihood function as

$$
\begin{aligned}
&D(\xi_{00}, \xi_{01}, \xi_{10}, \xi_{11}, f_1, f_2 \mid \hat{\xi}_{00}^{(t)}, \hat{\xi}_{01}^{(t)}, \hat{\xi}_{10}^{(t)}, \hat{\xi}_{11}^{(t)}, \hat{f}_1^{(t)}, \hat{f}_2^{(t)}) \\
=&\mathbb{E}_{\boldsymbol{\theta}_1, \boldsymbol{\theta}_2 \mid \hat{\xi}_{00}^{(t)}, \hat{\xi}_{01}^{(t)}, \hat{\xi}_{10}^{(t)}, \hat{\xi}_{11}^{(t)}, \hat{f}_1^{(t)}, \hat{f}_2^{(t)}}[l(\boldsymbol{p}_1, \boldsymbol{p}_2, \boldsymbol{\theta}_1, \boldsymbol{\theta}_2)] \\
=&\sum_{i=1}^{m} [(\gamma_{i,00}^{(t)} + \gamma_{i,01}^{(t)}) \log f_0(p_{1i}) + (\gamma_{i,10}^{(t)} + \gamma_{i,11}^{(t)}) \log f_1(p_{1i}) + (\gamma_{i,00}^{(t)} + \gamma_{i,10}^{(t)}) \log f_0(p_{2i}) \\
&+ (\gamma_{i,01}^{(t)} + \gamma_{i,11}^{(t)}) \log f_2(p_{2i})] + \sum_{i=1}^{m} [\gamma_{i,00}^{(t)} \log \xi_{00} + \gamma_{i,01}^{(t)} \log \xi_{01} + \gamma_{i,10}^{(t)} \log \xi_{10} + \gamma_{i,11}^{(t)} \log \xi_{11}].
\end{aligned}
$$

**M-step:** Update $(\hat{\xi}_{00}^{(t+1)}, \hat{\xi}_{01}^{(t+1)}, \hat{\xi}_{10}^{(t+1)}, \hat{\xi}_{11}^{(t+1)}, \hat{f}_1^{(t+1)}, \hat{f}_2^{(t+1)})$ by maximizing the conditional expectation of the log-likelihood function $D(\xi_{00}, \xi_{01}, \xi_{10}, \xi_{11}, f_1, f_2 \mid \hat{\xi}_{00}^{(t)}, \hat{\xi}_{01}^{(t)}, \hat{\xi}_{10}^{(t)}, \hat{\xi}_{11}^{(t)}, \hat{f}_1^{(t)}, \hat{f}_2^{(t)})$ subject to constraint $\xi_{00} + \xi_{01} + \xi_{10} + \xi_{11} = 1$. We obtain

$$
\begin{aligned}
\hat{\xi}_{00}^{(t+1)} &= \frac{\sum_{i=1}^{m} \gamma_{i,00}^{(t)}}{m}, \\
\hat{\xi}_{01}^{(t+1)} &= \frac{\sum_{i=1}^{m} \gamma_{i,01}^{(t)}}{m}, \\
\hat{\xi}_{10}^{(t+1)} &= \frac{\sum_{i=1}^{m} \gamma_{i,10}^{(t)}}{m}, \\
\hat{\xi}_{11}^{(t+1)} &= \frac{\sum_{i=1}^{m} \gamma_{i,11}^{(t)}}{m},
\end{aligned}
$$

and

$$
\hat{f}_1^{(t+1)} = \arg\max_{\tilde{f}_1 \in \mathbb{H}} \left\{ \sum_{i=1}^{m} (\gamma_{i,10}^{(t)} + \gamma_{i,11}^{(t)}) \log \tilde{f}_1(p_{1i}) \right\}, \tag{S1}
$$

$$
\hat{f}_2^{(t+1)} = \arg\max_{\tilde{f}_2 \in \mathbb{H}} \left\{ \sum_{i=1}^{m} (\gamma_{i,01}^{(t)} + \gamma_{i,11}^{(t)}) \log \tilde{f}_2(p_{2i}) \right\}, \tag{S2}
$$

where $\mathbb{H}$ is the set of $p$-value density functions under the non-null. We repeat the above **E-step** and **M-step** until the algorithm converges.

Next we provide specific steps to solve (S1) and (S2) using the Pool-Adjacent-Violators Algorithm (PAVA) [2] under the monotone likelihood ratio assumption [3, 4, 5]. Denote $Q_{1i}^{(t)} = \gamma_{i,10}^{(t)} + \gamma_{i,11}^{(t)}$ and $Q_{2i}^{(t)} = \gamma_{i,01}^{(t)} + \gamma_{i,11}^{(t)}, i = 1, \ldots, m$. Let $0 = p_{1(0)} \leq p_{1(1)} \leq \cdots \leq p_{1(m)}$ be the order statistics of $\boldsymbol{p}_1$ and denote $Q_{1(i)}^{(t)}$ as corresponding $Q_{1i}^{(t)}$ associated with $p_{1(i)}$. Let $0 = p_{2(0)} \leq p_{2(1)} \leq \cdots \leq p_{2(m)}$ be the order statistics of $\boldsymbol{p}_2$ and denote $Q_{2(i)}^{(t)}$ as corresponding $Q_{2i}^{(t)}$ associated with $p_{2(i)}$. Define $y_{1i} = f_1\left(p_{1(i)}\right), \boldsymbol{y}_1 = (y_{11}, \ldots, y_{1m})$ and $y_{2i} = f_2\left(p_{2(i)}\right), \boldsymbol{y}_2 = (y_{21}, \ldots, y_{2m})$, we can write (S1) and (S2) as

$$\hat{\boldsymbol{y}}_1^{(t+1)} = \arg\max_{\boldsymbol{y}_1 \in \mathcal{M}_1} \left\{ \sum_{i=1}^m Q_{1(i)}^{(t)} \log y_{1i} \right\}, \text{ subject to } \sum_{i=1}^m y_{1i}(p_{1(i)} - p_{1(i-1)}) = 1,$$

$$\hat{\boldsymbol{y}}_2^{(t+1)} = \arg\max_{\boldsymbol{y}_2 \in \mathcal{M}_2} \left\{ \sum_{i=1}^m Q_{2(i)}^{(t)} \log y_{2i} \right\}, \text{ subject to } \sum_{i=1}^m y_{2i}(p_{2(i)} - p_{2(i-1)}) = 1,$$

where $\mathcal{M}_1 = \{(y_{11}, \ldots, y_{1m}) : y_{11} \geq \cdots \geq y_{1m} \geq 0\}$ and $\mathcal{M}_2 = \{(y_{21}, \ldots, y_{2m}) : y_{21} \geq \cdots \geq y_{2m} \geq 0\}$.

By the Lagrangian multiplier, the objective functions we want to maximize are

$$\sum_{i=1}^m Q_{1(i)}^{(t)} \log y_{1i} + \lambda_1 \left\{ \sum_{i=1}^m y_{1i}(p_{1(i)} - p_{1(i-1)}) - 1 \right\},$$

$$\sum_{i=1}^m Q_{2(i)}^{(t)} \log y_{2i} + \lambda_2 \left\{ \sum_{i=1}^m y_{2i}(p_{2(i)} - p_{2(i-1)}) - 1 \right\}.$$

Taking derivatives with respect to $y_{1i}, \lambda_1$ and $y_{2i}, \lambda_2$, respectively, we have estimates

$$\hat{\lambda}_1 = -\sum_{i=1}^m Q_{1(i)}^{(t)}, \;\; \tilde{y}_{1i} = \frac{Q_{1(i)}^{(t)}}{Q_1^{(t)}(p_{1(i)} - p_{1(i-1)})},$$

$$\hat{\lambda}_2 = -\sum_{i=1}^m Q_{2(i)}^{(t)}, \;\; \tilde{y}_{2i} = \frac{Q_{2(i)}^{(t)}}{Q_2^{(t)}(p_{2(i)} - p_{2(i-1)})},$$

where $Q_1^{(t)} = \sum_{i=1}^m Q_{1(i)}^{(t)}$ and $Q_2^{(t)} = \sum_{i=1}^m Q_{2(i)}^{(t)}$.

To incorporate the monotone constraints on $y_{1i}$ and $y_{2i}$, we minimize

$$\sum_{i=1}^m \left\{ -Q_{1(i)}^{(t)} \log y_{1i} + Q_1^{(t)} (p_{1(i)} - p_{1(i-1)}) y_{1i} \right\} = \sum_{i=1}^m Q_{1(i)}^{(t)} \left\{ -\log y_{1i} - \frac{-Q_1^{(t)} \left(p_{1(i)} - p_{1(i-1)}\right)}{Q_{1(i)}^{(t)}} y_{1i} \right\},$$

subject to $y_{11} \geq \cdots \geq y_{1m}$, and minimize

$$\sum_{i=1}^m \left\{ -Q_{2(i)}^{(t)} \log y_{2i} + Q_2^{(t)} (p_{2(i)} - p_{2(i-1)}) y_{2i} \right\} = \sum_{i=1}^m Q_{2(i)}^{(t)} \left\{ -\log y_{2i} - \frac{-Q_2^{(t)} \left(p_{2(i)} - p_{2(i-1)}\right)}{Q_{2(i)}^{(t)}} y_{2i} \right\},$$

subject to $y_{21} \geq \cdots \geq y_{2m}$.

Let

$$(\hat{u}_{11}, \ldots, \hat{u}_{1m}) = \arg \min_{u_{11}, \ldots, u_{1m}} \sum_{i=1}^m Q_{1(i)}^{(t)} \left( u_{1i} - \frac{-Q_1^{(t)} \left(p_{1(i)} - p_{1(i-1)}\right)}{Q_{1(i)}^{(t)}} \right)^2$$

subject to $u_{11} \geq u_{12} \geq \cdots \geq u_{1m}$, and

$$(\hat{u}_{21}, \ldots, \hat{u}_{2m}) = \arg \min_{u_{21}, \ldots, u_{2m}} \sum_{i=1}^m Q_{2(i)}^{(t)} \left( u_{2i} - \frac{-Q_2^{(t)} \left(p_{2(i)} - p_{2(i-1)}\right)}{Q_{2(i)}^{(t)}} \right)^2$$

subject to $u_{21} \geq u_{22} \geq \cdots \geq u_{2m}$. The solutions take the max-min form

$$\hat{u}_{1i} = \max_{b \geq i} \min_{a \leq i} \frac{-Q_1^{(t)} \sum_{k=a}^b \left(p_{1(k)} - p_{1(k-1)}\right)}{\sum_{k=a}^b Q_{1(k)}^{(t)}},$$
$$\hat{u}_{2i} = \max_{b \geq i} \min_{a \leq i} \frac{-Q_2^{(t)} \sum_{k=a}^b \left(p_{2(k)} - p_{2(k-1)}\right)}{\sum_{k=a}^b Q_{2(k)}^{(t)}},$$

which can be obtained by PAVA [2]. Our final estimates are given by $\hat{y}_{1i} = -\frac{1}{\hat{u}_{1i}}$ and $\hat{y}_{2i} = -\frac{1}{\hat{u}_{2i}}$ for $i = 1, \ldots, m$ according to Theorem 3.1 of [6].

# B Competing methods

In the simulation studies, we compared STAREG to several replicability analysis methods, including *ad hoc* BH, MaxP [7, 8], MaRR [9], radjust [10] and JUMP [11]. Let $(p_{1i}, p_{2i}), i = 1, \dots, m$ denote the paired $p$-values from two studies. We review these competing methods as follows.

## B.1 The *ad hoc* BH method

BH [12] is the most popular multiple testing procedure that controls the FDR for $m$ independent or positively correlated tests. In study $j, j = 1, 2$, the BH procedure proceeds as below:

- *Step 1.* Let $p_{j(1)} \leq p_{j(2)} \leq \cdots \leq p_{j(m)}$ be the ordered $p$-values in study $j$, and denote by $H_{(i)}^{(j)}$ the null hypothesis corresponding to $p_{j(i)}$;
- *Step 2.* Find the largest $i$ such that $p_{j(i)} \leq \frac{i}{m}\alpha$, i.e., $\hat{k} = \max\{i \geq 1 : p_{j(i)} \leq \frac{i}{m}\alpha\}$, and $\hat{k} = 0$ if the set is empty;
- *Step 3.* Reject all $H_{(i)}^{(j)}$ for $i = 1, \dots, \hat{k}$.

The *ad hoc* BH method for replicability analysis identifies features rejected by both studies as replicable signals.

## B.2 The MaxP method

Define the maximum $p$-values as

$$q_i = \max\{p_{1i}, p_{2i}\}, i = 1, \dots, m.$$

As discussed in [11], $q_i$ follows a super-uniform distribution under the composite null. The naïve MaxP method directly applies BH [12] to $q_i, i = 1, \ldots, m$ for replicability analysis.

## B.3 The JUMP procedure

### B.3.1 The original JUMP procedure applied to two studies

The JUMP procedure [11] works on the maximum of $p$-values across two studies. Define

$$p_i^{\max} = \max\{p_{1i}, p_{2i}\}, i = 1, \ldots, m.$$

Let $s_i = (\theta_{1i}, \theta_{2i})$, $i = 1, \ldots, m$ denote the joint hidden states across two studies. Then $s_i \in \{(0,0), (0,1), (1,0), (1,1)\}$ with $\mathbb{P}(s_i = (k,l)) = \xi_{kl}$ for $k, l = 0, 1$ and $\sum_{k,l} \xi_{kl} = 1$. It can be shown that

$$\begin{aligned}
&\mathbb{P}\left(p_i^{\max} \le t \mid H_{i0} \text{ is true}\right) \\
=&\frac{\xi_{00}\mathbb{P}(p_i^{\max} \le t \mid s_i = (0,0))}{\xi_{00} + \xi_{01} + \xi_{10}} + \frac{\xi_{01}\mathbb{P}(p_i^{\max} \le t \mid s_i = (0,1))}{\xi_{00} + \xi_{01} + \xi_{10}} + \frac{\xi_{10}\mathbb{P}(p_i^{\max} \le t \mid s_i = (1,0))}{\xi_{00} + \xi_{01} + \xi_{10}} \\
\le&\frac{\xi_{00}t^2 + (\xi_{01} + \xi_{10})t}{\xi_{00} + \xi_{01} + \xi_{10}} \le t,
\end{aligned}$$

which means that $p_i^{\max}$ follows a super-uniform distribution under the replicability null. Denote

$$G(t) = \frac{\xi_{00}t^2 + (\xi_{01} + \xi_{10})t}{\xi_{00} + \xi_{01} + \xi_{10}}.$$

For a given threshold $t \in (0, 1)$, a conservative estimate of the FDR is obtained by

$$\text{FDR}^*(t) = \frac{m(\xi_{00} + \xi_{01} + \xi_{10})G(t)}{\sum_{i=1}^{m} I\{p_i^{\max} \le t\} \vee 1}.$$

Following [13, 14], the proportion of null hypotheses in study $j$ can be estimated by

$$\hat{\pi}_0^{(j)}(\lambda_j) = \frac{\sum_{i=1}^m I\{p_{ji} \geq \lambda_j\}}{m(1-\lambda_j)}, \quad j = 1, 2.$$

Similarly, $\xi_{00}$ is estimated by

$$\hat{\xi}_{00}(\lambda_3) = \frac{\sum_{i=1}^m I\{p_{1i} \geq \lambda_3, p_{2i} \geq \lambda_3\}}{m(1-\lambda_3)^2},$$

where $\lambda_1, \lambda_2$ and $\lambda_3$ are tuning parameters that can be selected by using the smoothing method provided in [15]. Then we have

$$\hat{\xi}_{01} = \hat{\pi}_0^{(1)} - \hat{\xi}_{00}, \quad \hat{\xi}_{10} = \hat{\pi}_0^{(2)} - \hat{\xi}_{00}.$$

With these estimates, we have a plug-in estimate of FDR,

$$\widehat{\mathrm{FDR}}^*(t) = \frac{m(\hat{\xi}_{00}t^2 + \hat{\xi}_{01}t + \hat{\xi}_{10}t)}{\sum_{i=1}^m I\{p_i^{\max} \leq t\} \vee 1}.$$

The JUMP procedure works as follows.

- *Step 1.* Let $p_{(1)}^{\max} \leq \cdots \leq p_{(m)}^{\max}$ be the ordered maximum of $p$-values and denote by $H_{(i)}$ the corresponding hypothesis;

- *Step 2.* Find the largest $k$ such that the estimated FDR is controlled, i.e.,

$$\hat{k} = \max\{1 \leq k \leq m : \widehat{\mathrm{FDR}}^*(p_{(k)}^{\max}) \leq \alpha\};$$

- *Step 3.* Reject $H_{(i)}$, $i = 1, \ldots, \hat{k}$.

### B.3.2 Extending the JUMP procedure to three studies

When we have three studies with $p$-values, $(p_{1i}, p_{2i}, p_{3i}), i = 1, \dots, m$, define the maximum of $p$-values as

$$p_i^{\max} = \max\{p_{1i}, p_{2i}, p_{3i}\}, i = 1, \dots, m.$$

Let $s_i = (\theta_{1i}, \theta_{2i}, \theta_{3i}), i = 1, \dots, m$ denote the joint hidden states across three studies with prior probabilities $P(s_i = (k, l, r)) = \xi_{klr}$, where $k, l, r = 0, 1$ and $\sum_{k,l,r} \xi_{klr} = 1$, $s_i \in \{(0,0,0), (0,0,1), (0,1,0), (0,1,1), (1,0,0), (1,0,1), (1,1,0), (1,1,1)\}$. The replicability null hypothesis of three studies is given by

$$H_{i0} : s_i \in \mathbb{H} = \{(0,0,0), (0,0,1), (0,1,0), (0,1,1), (1,0,0), (1,0,1), (1,1,0)\}, i = 1, \dots, m.$$

It can be shown that

$$\begin{aligned}
\mathbb{P}(p_i^{\max} \le t | H_{0i} \text{ is true}) &= \frac{\mathbb{P}(p_i^{\max} \le t, H_{i0} \text{ is true})}{\mathbb{P}(H_{i0} \text{ is true})} \\
&= \frac{\sum_{(k,l,r)\in\mathbb{H}} \xi_{klr} \mathbb{P}(p_i^{\max} \le t | s_i = (k,l,r))}{\sum_{(k,l,r)\in\mathbb{H}} \xi_{klr}} \\
&\le \frac{\xi_{000} t^3 + (\xi_{001} + \xi_{010} + \xi_{100}) t^2 + (\xi_{011} + \xi_{101} + \xi_{110}) t}{\sum_{(k,l,r)\in\mathbb{H}} \xi_{klr}} \le t,
\end{aligned}$$

which means the $p_i^{\max}$ follows a super-uniform distribution under the replicability null. Denote

$$G(t) = \xi_{000} t^3 + (\xi_{001} + \xi_{010} + \xi_{100}) t^2 + (\xi_{011} + \xi_{101} + \xi_{110}) t.$$

For a given threshold $t \in (0,1)$, a conservative estimate of the FDR is obtained by

$$\text{FDR}^*(t) = \frac{mG(t)}{\sum_{i=1}^m I\{p_i^{\max} \le t\} \vee 1}.$$

Following [13, 14], the proportion of null hypotheses in study $j$ can be estimated by

$$\hat{\pi}_0^{(j)}(\lambda_j) = \frac{\sum_{i=1}^m I\{p_{ji} \ge \lambda_j\}}{m(1-\lambda_j)}, \quad j = 1, 2, 3.$$

Similarly,

$$\begin{aligned}
\hat{\xi}_{000}(\lambda_4) + \hat{\xi}_{001}(\lambda_4) &= \frac{\#\{p_{1i} \ge \lambda_4, p_{2i} \ge \lambda_4, i = 1, \dots, m\}}{m(1-\lambda_4)^2} \\
\hat{\xi}_{000}(\lambda_5) + \hat{\xi}_{010}(\lambda_5) &= \frac{\#\{p_{1i} \ge \lambda_5, p_{3i} \ge \lambda_5, i = 1, \dots, m\}}{m(1-\lambda_5)^2} \\
\hat{\xi}_{000}(\lambda_6) + \hat{\xi}_{100}(\lambda_6) &= \frac{\#\{p_{2i} \ge \lambda_6, p_{3i} \ge \lambda_6, i = 1, \dots, m\}}{m(1-\lambda_6)^2} \\
\hat{\xi}_{000}(\lambda_7) &= \frac{\#\{p_{1i} \ge \lambda_7, p_{2i} \ge \lambda_7, p_{3i} \ge \lambda_7, i = 1, \dots, m\}}{m(1-\lambda_7)^3},
\end{aligned}$$

where $\lambda_1, \dots, \lambda_7$ are tuning parameters that can be selected by using the smoothing method provided in [15]. Then we have

$$\begin{aligned}
\hat{\xi}_{011} &= \hat{\pi}_0^{(1)} - \hat{\xi}_{000} - \hat{\xi}_{001} - \hat{\xi}_{010}, \\
\hat{\xi}_{101} &= \hat{\pi}_0^{(2)} - \hat{\xi}_{000} - \hat{\xi}_{001} - \hat{\xi}_{100}, \\
\hat{\xi}_{110} &= \hat{\pi}_0^{(3)} - \hat{\xi}_{000} - \hat{\xi}_{010} - \hat{\xi}_{100}.
\end{aligned}$$

With these esitmates, we have a plug-in estimate of FDR,

$$\widehat{\mathrm{FDR}}^*(t) = \frac{m\hat{G}(t)}{\sum_{i=1}^m I\{p_i^{\max} \le t\} \vee 1},$$

where $\hat{G}(t) = \hat{\xi}_{000} t^3 + (\hat{\xi}_{001} + \hat{\xi}_{010} + \hat{\xi}_{100}) t^2 + (\hat{\xi}_{011} + \hat{\xi}_{101} + \hat{\xi}_{110}) t$.

Then the JUMP procedure can be applied to control the FDR for the replicability analysis across three studies.

## B.4 The MaRR procedure

The MaRR procedure [9] uses the maximum rank of each feature. The null hypothesis is that $H_{i0}$ : $p_{1i}$ and $p_{2i}$ are irreproducible. Denote $(R_{1i}, R_{2i})$ as the ranks of $(p_{1i}, p_{2i}), i = 1, \dots, m$ within each study. Define

$$M_i = \max\{R_{1i}, R_{2i}\}, i = 1, \dots, m.$$

Let $\pi_1$ denote the proportion of replicable signals. Under the assumptions:

(I1) if gene $g$ is reproducible and gene $h$ is irreproducible

$$R_{1g} < R_{1h}, \quad R_{2g} < R_{2h};$$

(I2) the correlation between the ranks of the reproducible gene is non-negative;

(I3) the two ranks of the irreproducible gene are independent,

irreproducible ranks $R_{1i}$ and $R_{2i}$ are uniformly distributed between $\lfloor m\pi_1 \rfloor + 1$ and $m$. Denote the conditional null survival function of $M_i/m$ as

$$\begin{aligned} S_{m,\pi_1}(x) &= P(M_i/m > x \mid \text{ gene } i \text{ is irreproducible}) \\ =& 1 - P\left(R_{1i}/m \leq x, R_{2i}/m \leq x \mid \text{ gene } i \text{ is irreproducible}\right) \\ =& 1 - \prod_{j=1}^{2} P\left(R_{ji}/m \leq x \mid \text{ gene } i \text{ is irreproducible}\right) \\ =& \begin{cases} 1, & x < \pi_1, \\ 1 - \frac{(i_x - j_{\pi_1})^2}{(m - j_{\pi_1})^2}, & \pi_1 \leq x \leq 1, \end{cases} \end{aligned}$$

where $i_x = \lfloor mx \rfloor$ and $j_{\pi_1} = \lfloor m\pi_1 \rfloor$. The limiting conditional survival function of $M_i/m$ under the null is

$$S_{m,\pi_1}(x) \to S_{\pi_1}(x) = \begin{cases} 1 & x < \pi_1 \\ 1 - \frac{(x-\pi_1)^2}{(1-\pi_1)^2} & \pi_1 \leq x \leq 1 \\ 0 & 1 < x \end{cases} \cdot$$

The empirical survival function can be estimated by $\hat{S}_m(x) = \frac{1}{m}\sum_{i=1}^{m} I(M_i/m \geq x)$, $x \in (0,1)$. By strong law of large numbers and Bayesian formula,

$$\begin{aligned} \hat{S}_m(x) \to & P(M_i/m \geq x) \\ & = (1-\pi_1)P(M_i/m \geq x \mid \text{ gene } i \text{ is irreproducible}) + \pi_1 \times 0 \\ & = (1-\pi_1)S_{\pi_1}(x) \text{ for } x \in (\pi_1, 1). \end{aligned}$$

If we estimate $\pi_1$ by $i/m$, we can define the mean square error (MSE) as follows.

$$\text{MSE}(i/m) = (m-i)^{-1}\sum_{j=i}^{m}\left(\hat{S}_m(j/m) - (1-i/m)S_{i/m}(j/m)\right)^2.$$

$\hat{k}$ is chosen to minimize the MSE in the range between 0 and $\lfloor 0.9m \rfloor$.

$$\hat{k} = \underset{i=0,1,\ldots,\lfloor 0.9m \rfloor}{\arg\min} \{\text{MSE}(i/m)\}.$$

Thus $\hat{k}/m$ serves as a good estimation of $\pi_1$. To control the FDR at level $\alpha$, the MaRR generates the rejection threshold as follows

$$\text{Define } \hat{N} = \max_{\hat{k} < i \leq n}\left\{ i : m\widehat{\text{FDR}}(i) = \frac{(i-\hat{k})^2}{Q(i)(m-\hat{k})} \leq \alpha \right\},$$

where $Q(i) = \sum_{j=1}^{m} I(M_j \leq i)$. Reject all features associated with $M_i \leq \hat{N}$. [9] relax assumption (I1) to (R1): $P(R_{1g} < R_{1h}) > 1/2$ and $P(R_{2g} < R_{2h}) > 1/2$, which is more plausible in practice.

## B.5 The radjust procedure

The radjust procedure [10] works as follows,

- *Step 1.* For a pre-specified FDR level $\alpha$, compute

$$R = \max\left[r : \sum_{i \in \mathcal{S}_1 \cap \mathcal{S}_2} I\left\{(p_{1i}, p_{2i}) \leq \left(\frac{r\alpha}{2|\mathcal{S}_2|}, \frac{r\alpha}{2|\mathcal{S}_1|}\right)\right\} = r\right],$$

  where $\mathcal{S}_j$ is the set of features pre-selected in study $j$ for $j = 1, 2$. By default, it selects features with $p$-values less than or equal to $\alpha/2$.

- *Step 2.* Declare features as replicable if they are in the following set

$$\mathcal{R} = \left\{i : (p_{1i}, p_{2i}) \leq \left(\frac{R\alpha}{2|\mathcal{S}_2|}, \frac{R\alpha}{2|S_1|}\right), i \in \mathcal{S}_1 \cap \mathcal{S}_2\right\}.$$

[10] also provides an adaptive version of the radjust procedure, which first estimates the fractions of true null hypotheses among the pre-selected features. The fractions in the two studies are estimated as follows.

$$\hat{\pi}_0^{(1)} = \frac{1 + \sum_{i \in \mathcal{S}_{2,\alpha}} I(p_{1i} > \alpha)}{|\mathcal{S}_{2,\alpha}|(1-\alpha)}, \quad \hat{\pi}_0^{(2)} = \frac{1 + \sum_{i \in \mathcal{S}_{1,\alpha}} I(p_{2i} > \alpha)}{|\mathcal{S}_{1,\alpha}|(1-\alpha)}, \tag{S3}$$

where $\mathcal{S}_{j,\alpha} = \mathcal{S}_j \cap \{1 \leq i \leq m : p_{ji} \leq \alpha\}$, $j = 1, 2$. The adaptive procedure with a nominal FDR level $\alpha$ works as follows.

- *Step 1.* Compute $\hat{\pi}_0^{(1)}$ and $\hat{\pi}_0^{(2)}$ using (S3). Let

$$R = \max\left[ r : \sum_{i \in \mathcal{S}_{1,\alpha} \cap \mathcal{S}_{2,\alpha}} I\left\{ (p_{1i}, p_{2i}) \leq \left( \frac{r\alpha}{2|\mathcal{S}_{2,\alpha}|\hat{\pi}_0^{(1)}}, \frac{r\alpha}{2|\mathcal{S}_{1,\alpha}|\hat{\pi}_0^{(2)}} \right) \right\} = r \right],$$

- *Step 2.* Reject features with indices in the set

$$\mathcal{R} = \left\{ i : (p_{1i}, p_{2i}) \leq \left( \frac{R\alpha}{2|\mathcal{S}_{2,\alpha}|\hat{\pi}_0^{(1)}}, \frac{R\alpha}{2|S_{1,\alpha}|\hat{\pi}_0^{(2)}} \right), i \in \mathcal{S}_{1,\alpha} \cap \mathcal{S}_{2,\alpha} \right\}.$$

# C Additional simulations

## C.1 Simulations with dependent data

we simulated data with local dependence to examine the robustness of the STAREG. Specifically, in each study, we divided the $m = 10,000$ genes into 100 blocks and each block was further divided into two sub-blocks of equal size. Within each sub-block, there is a constant positive correlation (e.g., 0.2) and a constant negative correlation (e.g., $-0.2$), respectively. Data in different blocks are independent. In each block of study $j$ ($j = 1, 2$), the corresponding hidden states are vector $\boldsymbol{\theta}_j^* = (\theta_{jk}^*)_{k=1}^{100}$, the test statistics were generated from a 100-dimensional multivariate normal distribution: $\boldsymbol{X}_j \sim \text{MVN}(\boldsymbol{\mu}, \Sigma)$, where the mean vector $\boldsymbol{\mu} = \boldsymbol{\theta}_j^* \cdot \mu_j$. We use a 4-dimensional covariance matrix with $\rho = 0.2$ to illustrate the pattern of $\Sigma$,

$$\Sigma = \begin{pmatrix} 1 & 0.2 & -0.2 & -0.2 \\ 0.2 & 1 & -0.2 & -0.2 \\ -0.2 & -0.2 & 1 & 0.2 \\ -0.2 & -0.2 & 0.2 & 1 \end{pmatrix}.$$

We set $m = 10,000, \xi_{01} = \xi_{10}$ and $\xi_{11} = 0.05$, and examined the performance of different

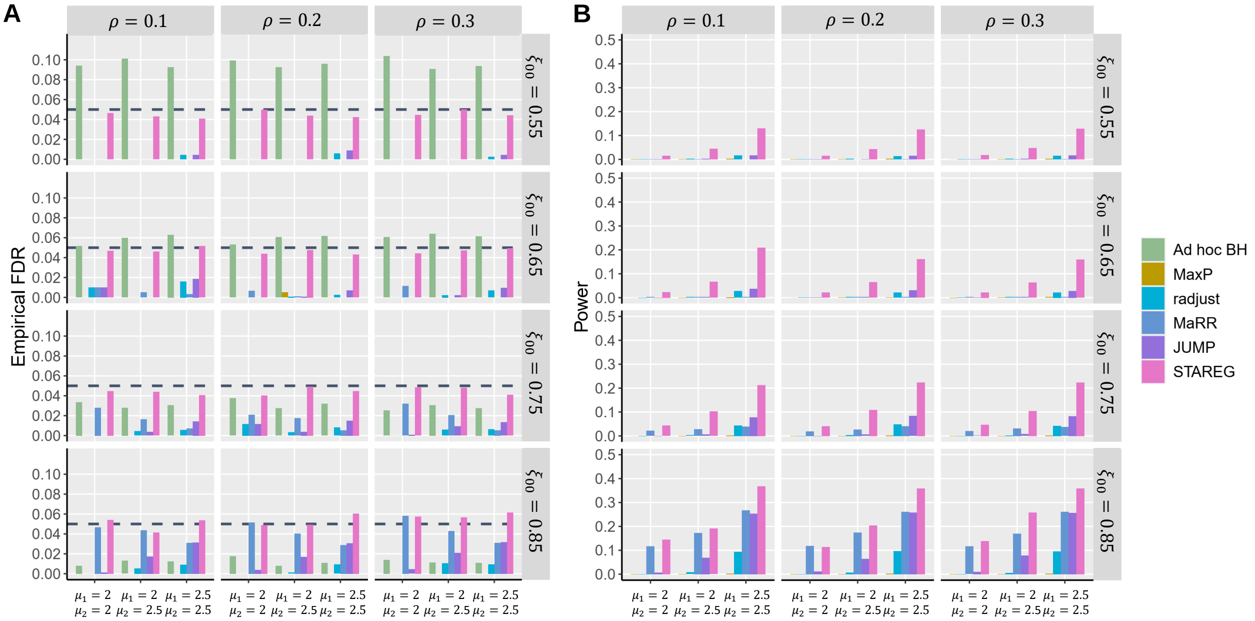

Fig S1: Simulation results of FDR and power for dependent data. Simulations were performed with $m = 10,000, \xi_{11} = 0.05$ and $\xi_{01} = \xi_{10}$ under different $\mu_1, \mu_2, \xi_{00}$ and $\rho$. The horizontal dashed line represents the target FDR level of 0.05. We do not present the power of *ad hoc* BH as it fails to control FDR.

methods under different $\mu_1, \mu_2, \xi_{00}$ and $\rho$. The FDR control and power comparisons at the FDR level 0.05 with different methods are presented in Fig S1. Fig S2 presents the FDR control and power comparison of different methods with varied FDR level when $m = 10,000, \xi_{00} = 0.95, \xi_{11} = 0.02$, $\xi_{01} = \xi_{10} = 0.015, \mu_1 = \mu_2 = 2$ and $\rho = 0.2$. In Fig S1A, the horizontal dashed lines indicate the target FDR level 0.05. We observe that *ad hoc* BH and MaRR do not control the FDR. Other methods control the FDR across all settings, where MaxP is overly conservative. STAREG has higher power than competing methods.

## C.2 Realistic simulations based on SRT data

We performed realistic simulations based on Replicate 1 and Replicate 8 of the ST data from MOB with parameters inferred from SPARK [3]. In all realistic simulations, we used pre-specified $\xi_{00}, \xi_{01}, \xi_{10}$ and $\xi_{11}$ to generate hidden states $\boldsymbol{\theta}_1$ and $\boldsymbol{\theta}_2$ for $m = 10,000$ genes from

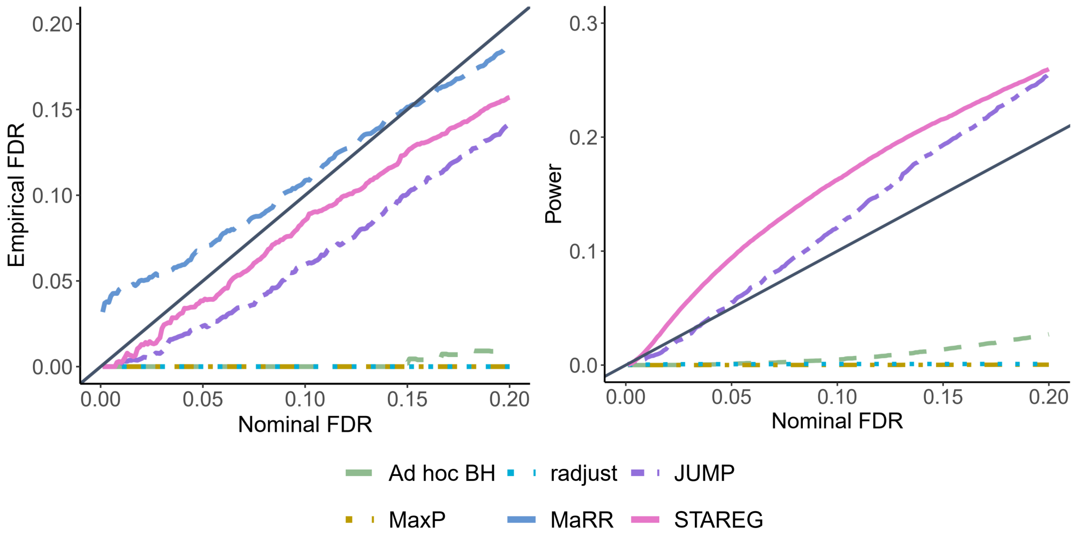


Fig S2: Simulation results of FDR and power for dependent data with varied nominal FDR. Simulaitons were performed with $m = 10,000, \xi_{00} = 0.95, \xi_{11} = 0.02$, $\xi_{01} = \xi_{10} = 0.015, \mu_1 = \mu_2 = 2$ and $\rho = 0.2$.

multinomial distribution. We then separately generated count data in two studies following the simulation strategy in [16] based on $\boldsymbol{\theta}_1$ and $\boldsymbol{\theta}_2$. Specifically, in study $j$ ($j = 1, 2$), for each gene, in turn, the read counts on the spot $i$ ($i = 1, \dots, n$, $n = 265$ for study 1 based on Replicate 1 and $n = 232$ for study 2 based on Replicate 8) were simulated from the following model:

$$y_i \sim \text{Poisson}(N_i \lambda_i), \ \log \lambda_i = \beta_i + \epsilon_i,$$

where $N_i$ denotes total read counts of all genes on the spot $i$, which can be obtained from data; $\lambda_i$ is the unknown relative expression level of the focal gene; the intercept $\beta_i$ represents the mean value of $\log \lambda_i$; and the error term $\epsilon_i \sim N(0, \tau_j^2)$ measures random noise independent of spatial locations, with the same variance across all spatial spots in the same study. The standard deviation $\tau_j$ for study $j$ was specified as $0.2, 0.5$, or $0.8$ in the simulations. For non-SVGs, we set the intercept $\beta_i$ to be constant across all spots and equal to the median of the intercepts estimated by SPARK in corresponding MOB replicates ($\beta_i = -10.46$ for study

1 and $\beta_i = -9.98$ for study 2). For SVGs, we introduced the spatial expression patterns by dividing the $n$ spots into two groups based on the three main patterns in MOB as illustrated in Fig 5A. In the low expression group, $\beta_i$ is set to be $-10.46$ for study 1 and $-9.98$ for study 2; and in the high expression group, $\beta_i$ is set to be two-fold (weak signal), three-fold (moderate signal) or four-fold (strong signal) higher than that in the low expression group on rate parameter scale, e.g., $e^{\beta_i} = 2 \cdot e^{-10.46}$ means $\beta_i$ is two-fold higher than $-10.46$. Finally, $\lambda_i$ can be simulated through $\lambda_i = e^{\beta_i + \epsilon_i}$, and $y_i$ was generated from the Poisson distribution with parameter $N_i \lambda_i$.

Simulated SRT data corresponding to three spatial expression patterns shown at the top of Fig S3 were generated in pairs and analyzed separately with SPARK to produce paired $p$-values for replicability analysis. Fig S3 presents the FDR control and power comparison of different methods for $m = 10,000$ genes at the target FDR level 0.05. We observe that the *ad hoc* BH failed to control FDR for the replicability null in some settings, while the FDR of all other methods can be controlled at the nominal level. MaRR is overly conservative when $\xi_{00} = 0.45$ or 0.65. STAREG demonstrated higher power across various settings. To have a fair comparison, we calculated empirical FDR, false positive rate, and power (true positive rate) with a range of FDR cutoffs from 0 to 1 based on different methods. The number of genes $m = 10,000$, and prior probabilities used to produce four hidden states were set as $\xi_{00} = 0.9, \xi_{01} = \xi_{10} = 0.025$, and $\xi_{11} = 0.05$. Overall, the simulation results in Fig S4 and S5 suggest that STAREG is more powerful than computing methods while controlling the FDR. The reason for the favorable performance of STAREG is due to the structure of the test statistic Lfdr, which efficiently combines information in both null and non-null and provides a better ranking of important genes compared with $p$-value-based and maximum rank-based methods, where only information contained in the null hypothesis is utilized. The heterogeneity of different studies manifest through $\xi_{01}$ and $\xi_{10}$. We model such heterogeneity through a four-group model expanding classic two-group model [17].

We evaluated the computational time of STAREG in simulation studies. As all methods we compared work directly with paired $p$-values, we only considered the computational overhead of replicability analysis after obtaining paired $p$-values. Prior probabilities used to produce four hidden states were set as $\xi_{00} = 0.9, \xi_{01} = \xi_{10} = 0.025$, and $\xi_{11} = 0.05$, the signal strengths were set to be strong for study 1 and moderate for study 2, and the noise levels in the two studies were $\tau_1 = \tau_2 = 0.2$. Table S1 summarizes the computational time of different methods for SRT data with different numbers of genes. All methods are implemented in R, in which STAREG uses Rcpp to speed up the computation. Computations were carried out in an i7-9750H 2.6GHz CPU with 64.0 GB RAM laptop. We observe that all methods are quick to compute. STAREG takes less time than JUMP and MaRR and longer time than *ad hoc* BH,

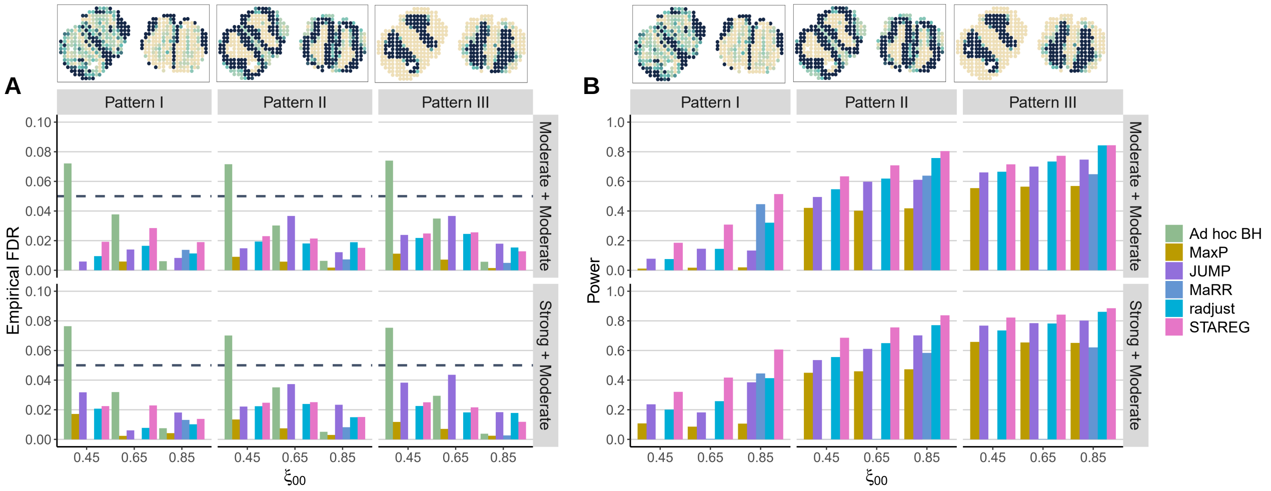


Fig S3: (**A**) FDR control of different methods in the realistic simulation studies based on SRT data [18]. (**B**) Power comparison of different methods in the realistic simulation studies based on SRT data and parameters. In each simulation, two SRT datasets were independently generated from corresponding MOB data with the same noise level ($\tau_1 = \tau_2 = 0.2$) and different prior probabilities (left: $\xi_{00} = 0.45, \xi_{01} = \xi_{10} = 0.05, \xi_{11} = 0.05$; middle: $\xi_{00} = 0.65, \xi_{01} = \xi_{10} = 0.15, \xi_{11} = 0.05$; right: $\xi_{00} = 0.85, \xi_{01} = \xi_{10} = 0.25, \xi_{11} = 0.05$). Each column corresponds to a different spatial expression pattern, as illustrated at the top (left to right: Pattern I-III). Each row corresponds to a different signal strength setting for the two studies (top: moderate signal for both studies; bottom: strong signal for study 1 and moderate signal for study 2). The horizontal dashed line represents the target FDR level of 0.05. We do not present the power of *ad hoc* BH as it fails to control FDR.

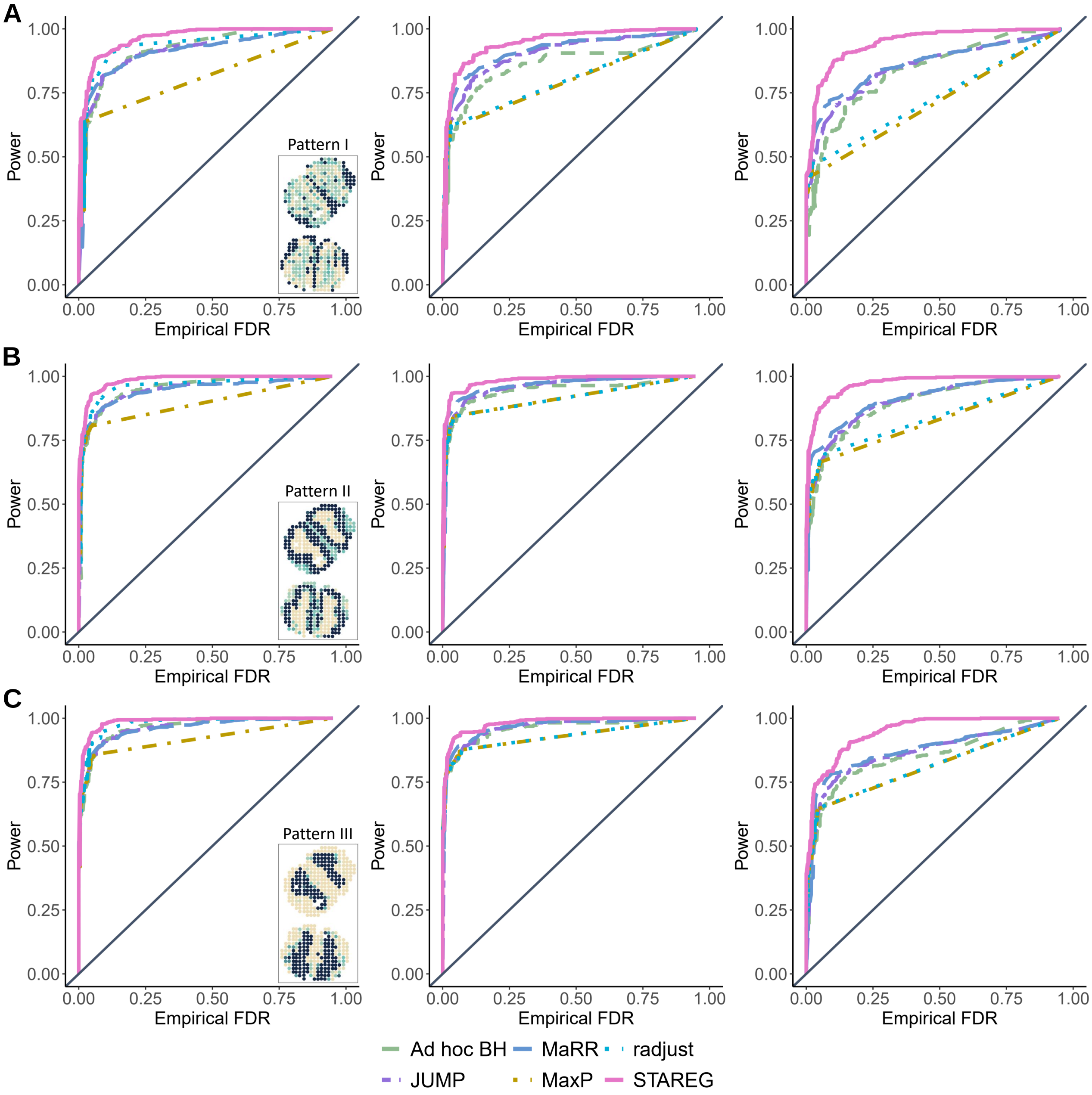


Fig S4: Plots of power (y-axis) over corresponding empirical FDR (x-axis) of different methods at a range of FDR cutoffs in the realistic simulation studies based on SRT data and parameters. Simulations were performed under the setting of $m = 10,000$, $\xi_{00} = 0.9, \xi_{01} = \xi_{10} = 0.025$ and $\xi_{11} = 0.05$. The signal strengths were set to be strong for study 1 and moderate for study 2 across all simulations. In each setting, two SRT datasets were independently generated from corresponding MOB replicates with different noise levels (top: $\tau_1 = 0.2, \tau_2 = 0.5$; middle: $\tau_1 = \tau_2 = 0.5$; bottom: $\tau_1 = 0.5, \tau_2 = 0.8$). Each row corresponds to a different spatial expression pattern, as illustrated in the panels. (**A**) Pattern I; (**B**) Pattern II; (**C**) Pattern III. The diagonal line with slope 1 is used as a reference.

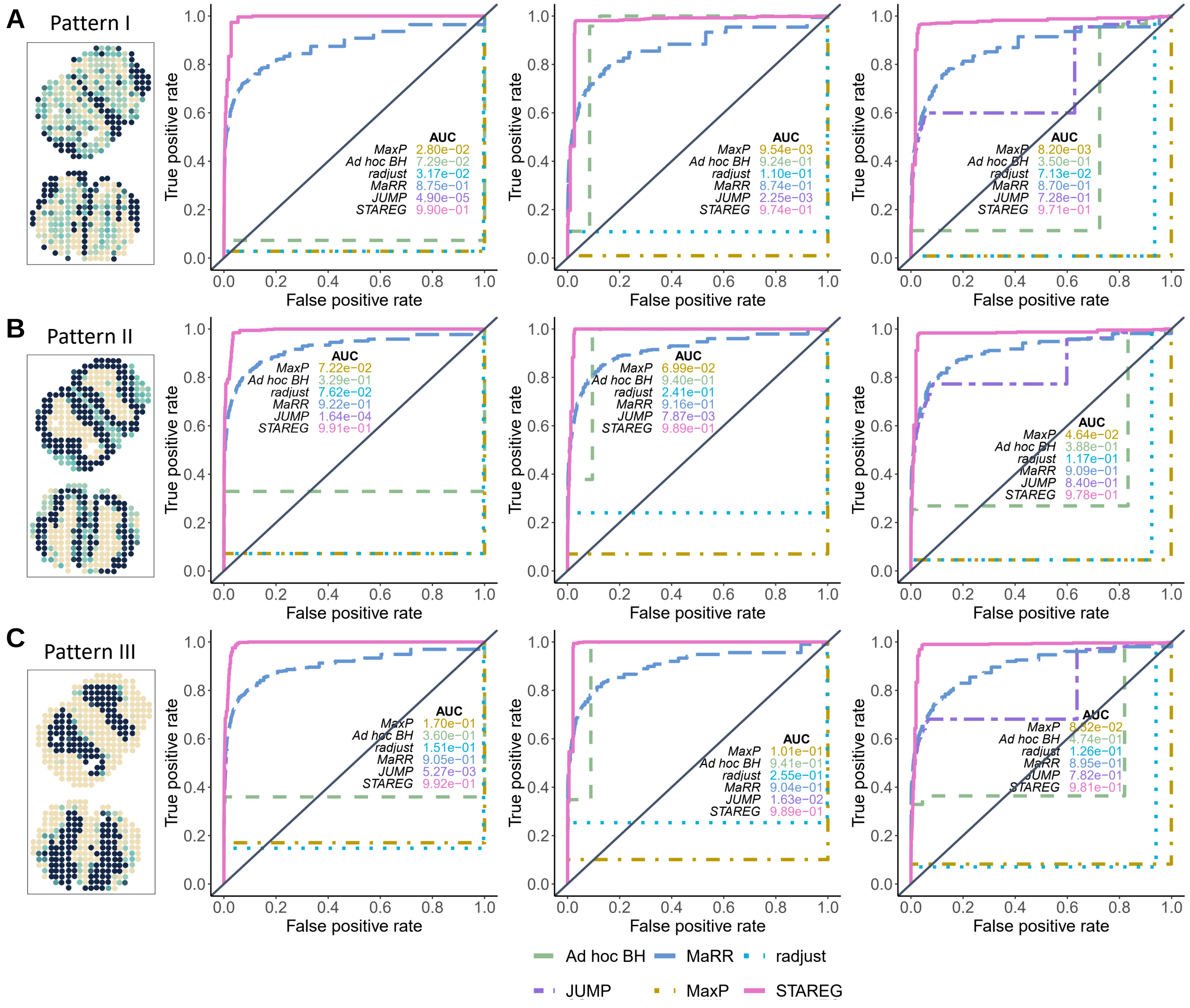


Fig S5: ROC curves and AUC values in the realistic simulation studies based on SRT data [18]. Simulations were performed under the setting of $m = 10,000, \xi_{00} = 0.9, \xi_{01} = \xi_{10} = 0.025$ and $\xi_{11} = 0.05$. The signal strengths were set to be strong for study 1 and weak for study 2 across all simulations. In each setting, two SRT datasets were independently generated from corresponding MOB replicates with different noise levels (top: $\tau_1 = \tau_2 = 0.2$; middle: $\tau_1 = 0.2, \tau_2 = 0.5$; bottom: $\tau_1 = \tau_2 = 0.5$). Each row corresponds to a different spatial expression pattern, as illustrated on the left. (**A**) Pattern I; (**B**) Pattern II; (**C**) Pattern III. The diagonal line with slope 1 is used as a reference.

MaxP, and radjust, though such differences can be ignored in practical data analysis.

Table S1: Computational time (in seconds) for replicability analysis in realistic simulation studies.

| Method \ # of genes | Pattern I 5,000 | Pattern I 10,000 | Pattern I 20,000 | Pattern II 5,000 | Pattern II 10,000 | Pattern II 20,000 | Pattern III 5,000 | Pattern III 10,000 | Pattern III 20,000 |
|---|---|---|---|---|---|---|---|---|---|
| *Ad hoc* BH | 0.0062 | 0.0064 | 0.0081 | 0.0056 | 0.0065 | 0.0109 | 0.0055 | 0.0057 | 0.0076 |
| MaxP | 0.0101 | 0.0140 | 0.0335 | 0.0108 | 0.0180 | 0.0362 | 0.0103 | 0.0152 | 0.0534 |
| JUMP | 0.0226 | 0.0480 | 0.0765 | 0.0287 | 0.0350 | 0.0670 | 0.0185 | 0.0191 | 0.0444 |
| MaRR | 0.5260 | 0.5734 | 7.6448 | 0.7240 | 1.9986 | 7.6125 | 0.5566 | 0.6881 | 7.5901 |
| radjust | 0.0114 | 0.0121 | 0.0159 | 0.0112 | 0.0133 | 0.0142 | 0.0112 | 0.0120 | 0.0145 |
| STAREG | 0.0167 | 0.0202 | 0.0497 | 0.0150 | 0.0249 | 0.0478 | 0.0154 | 0.0169 | 0.0364 |

# D Additional data analysis

## D.1 MOB data analysis using SPARK-X

For comparison, we used SPARK-X [19] to analyze the MOB data. After filtering out genes that are expressed in less than 10% of the spatial locations and selecting spatial locations with at least ten total read counts, we obtained $10,373$ genes on 265 spots in the Replicate 1 dataset and $9,671$ genes on 232 spots in Replicate 8 dataset. We applied SPARK-X separately to the two datasets to obtain two sequences of $p$-values. Next, we took paired $p$ values for $9,329$ genes common in both studies as input for the replicability analysis.

Using SPARK-X [19], the results of different methods at the FDR level 0.05 are summarized in Fig S6B. Overall, by using $p$-values generated by SPARK-X, all methods were less powerful than those using $p$-values generated by SPARK. STAREG detected 21 replicable SVGs, 7 of which were not identified by any other methods. All 21 replicable SVGs are identified by STAREG if we use $p$-values produced by SPARK. MaxP and JUMP identified 2 replicable SVGs, and radjust detected one more. MaRR and *ad hoc* BH identified 7 and 13 replicable SVGs, respectively. We clustered the 21 genes identified by STAREG into three

groups with distinct spatial expression patterns using R package *amap* v0.8-18. As shown in Fig S6A, the three distinct spatial patterns can be matched to three main layers in MOB: Pattern I corresponds to the granular cell layer, Pattern II corresponds to the mitral cell layer and Pattern III corresponds to the glomerular cell layer. We list spatial expression patterns of 7 replicable SVGs uniquely identified by STAREG in Fig S7 as additional evidence. We also calculated the Moran's $I$ statistic [20], a commonly used metric to quantify spatial autocorrelations. As shown in Fig S6C, the 7 replicable SVGs uniquely identified by STAREG have larger Moran's $I$ statistic than that of $9,308$ genes not identified by STARGE.

## D.2 Single-cell RNA-seq data analysis

We downloaded scRNA-seq data from two mouse aortic leukocyte samples measured with 10x chromium from PanglaoDB [21] (`https://panglaodb.se/samples.html`). We treat sample SRS2747908 as study 1, which includes read counts of $19,150$ genes in $1,629$ cells, and sample SRS2747908 as study 2, which includes count data of $21,052$ genes in $1,608$ cells. After filtering out genes expressed in less than 10 cells and selecting cells with at least $1,000$ total read counts, we obtained $10,512$ genes in 398 cells in study 1 and $12,868$ genes in $1,090$ cells in study 2. We used the cluster results published in PanglaoDB [21], resulting in 8 clusters with more than 10 cells in study 1 and 17 clusters with more than 10 cells in study 2, respectively. We further filtered out cells not in any of the above clusters, yielding 378 cells in Study 1 and $1,036$ cells in Study 2. We performed a differential analysis of T memory cells with the remaining cells using the Wilcox test [22], and got $p$-values. Next, we took common genes in both studies and obtained $10,274$ pairs of $p$-values as input for subsequent replicability analysis.

The results of different methods at the FDR level 0.01 are summarized in Fig S8. MaRR identified 447 replicable markers for T memory cells, MaxP detected an additional 265, and

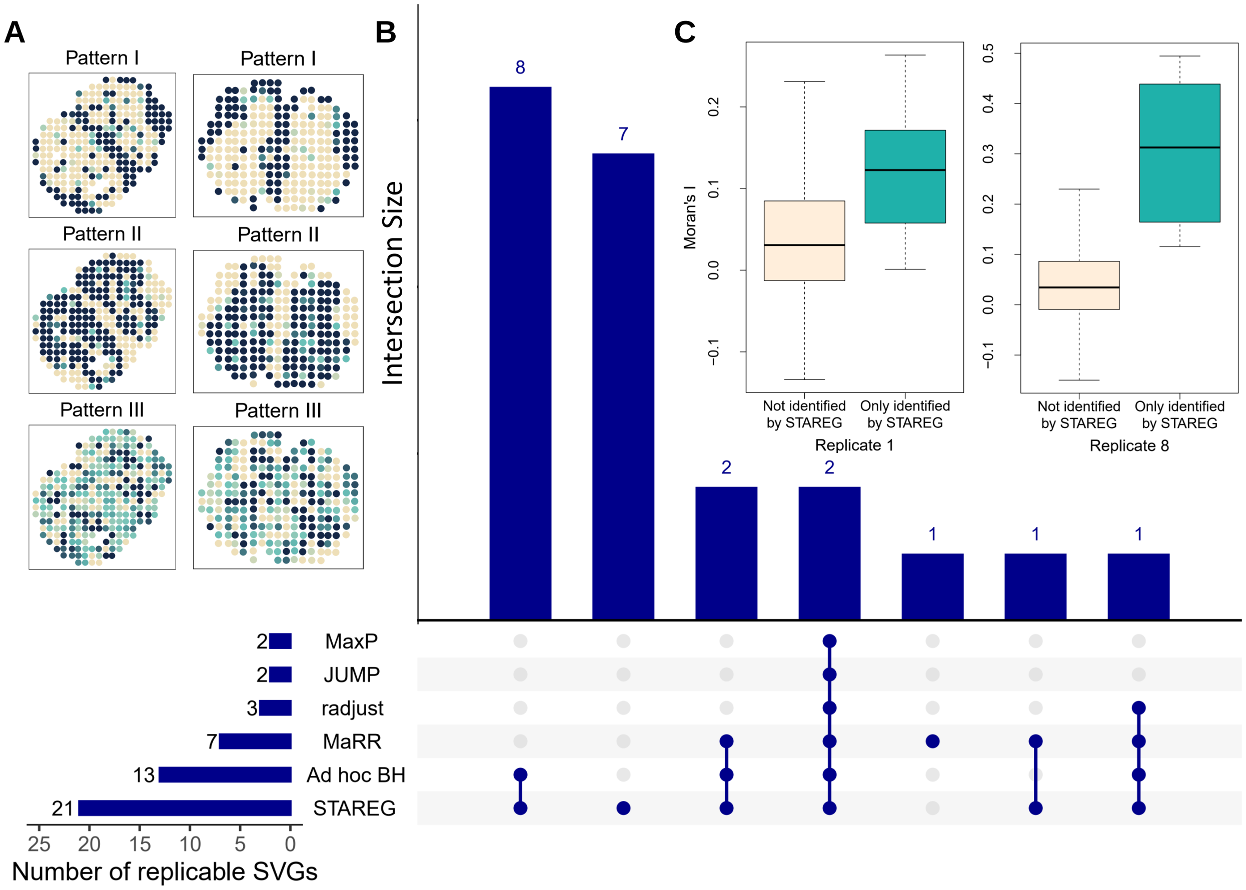


Fig S6: MOB data analysis results with SPARK-X at FDR level 0.05. (**A**) Three spatial patterns summarized based on 21 replicable SVGs identified by STAREG (left: ST Replicate 1 study; right: ST Replicate 8 study). (**B**) The number of replicable SVGs identified by different methods. (**C**) Moran's $I$ statistic of replicable SVGs only identified by STAREG compared to that of not replicable SVGs in the MOB data.

these 712 replicable markers were also detected by all other methods. *Ad hoc* BH identified 786 replicable markers, JUMP identified 881 replicable markers, and radjust identified 911 replicable markers. STAREG detected 1,093 replicable markers, including all those identified by other methods, and an additional 162 not detected by any other method. First, we used a publicly available database, PanglaoDB [21], to validate the results. Among the 162 replicable markers uniquely identified by STAREG, 30 were reported by PanlaoDB as differentially expressed in T memory cells in mouse aortic leukocytes (Table S2). Second, we used papers in

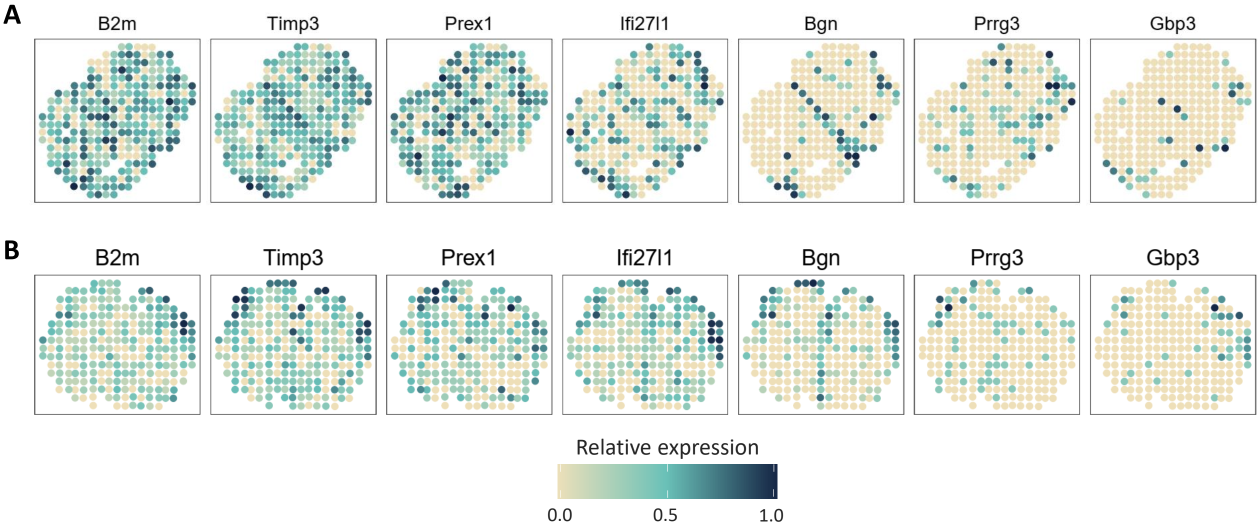


Fig S7: Spatial expression patterns of 7 replicable SVGs uniquely identified by STAREG with SPARK-X based on the (**A**) ST Replicate 1 data and (**B**) ST Replicate 8 data. Different colors represent relative gene expression levels (antique white: low; navy blue: high).

the literature to validate the remaining 132 markers, many of which have been demonstrated to be related to T memory cell functions. For instance, *Alcam* regulates multiple effector memory T cell functions via the *Cd6-Alcam* signaling pathway [23]; *Cd79a* is involved in a composite costimulatory domain *Cd79a/Cd40*, which enriches genes correlated with T-cell proliferation, interferon signaling pathway, naïve and memory T-cell signatures, and T-cell exhaustion [24]; *Trdv2-2* is a T cell receptor that can regulate T memory cells, and it influences the multiple fates of T cells in the memory pool by interacting with peptide/*MHC* [25, 26].

Table S2: The 30 validated replicable cell markers uniquely identified by STAREG.

| | | | | | | | | | |
|---|---|---|---|---|---|---|---|---|---|
| 1 | *Adgre5* | 7 | *Ctsb* | 13 | *Gnb2l1* | 19 | *Map3k1* | 25 | *Rpsaps10* |
| 2 | *Arl6ip5* | 8 | *Ddx5* | 14 | *Hnrnpab* | 20 | *Ppp1r11* | 26 | *Selplg* |
| 3 | *Bsg* | 9 | *Efhd2* | 15 | *Ict1* | 21 | *Rpl3* | 27 | *Sf3b2* |
| 4 | *Capzb* | 10 | *Fam49b* | 16 | *Il21r* | 22 | *Rpl36aps2* | 28 | *Smad7* |
| 5 | *Cited2* | 11 | *Fyb* | 17 | *Kif5b* | 23 | *Rps17* | 29 | *Tax1bp1* |
| 6 | *Clic1* | 12 | *Gm9493* | 18 | *Lsp1* | 24 | *Rpsa* | 30 | *Tpt1* |

To further examine the biological relevance of replicable markers, we performed a gene set

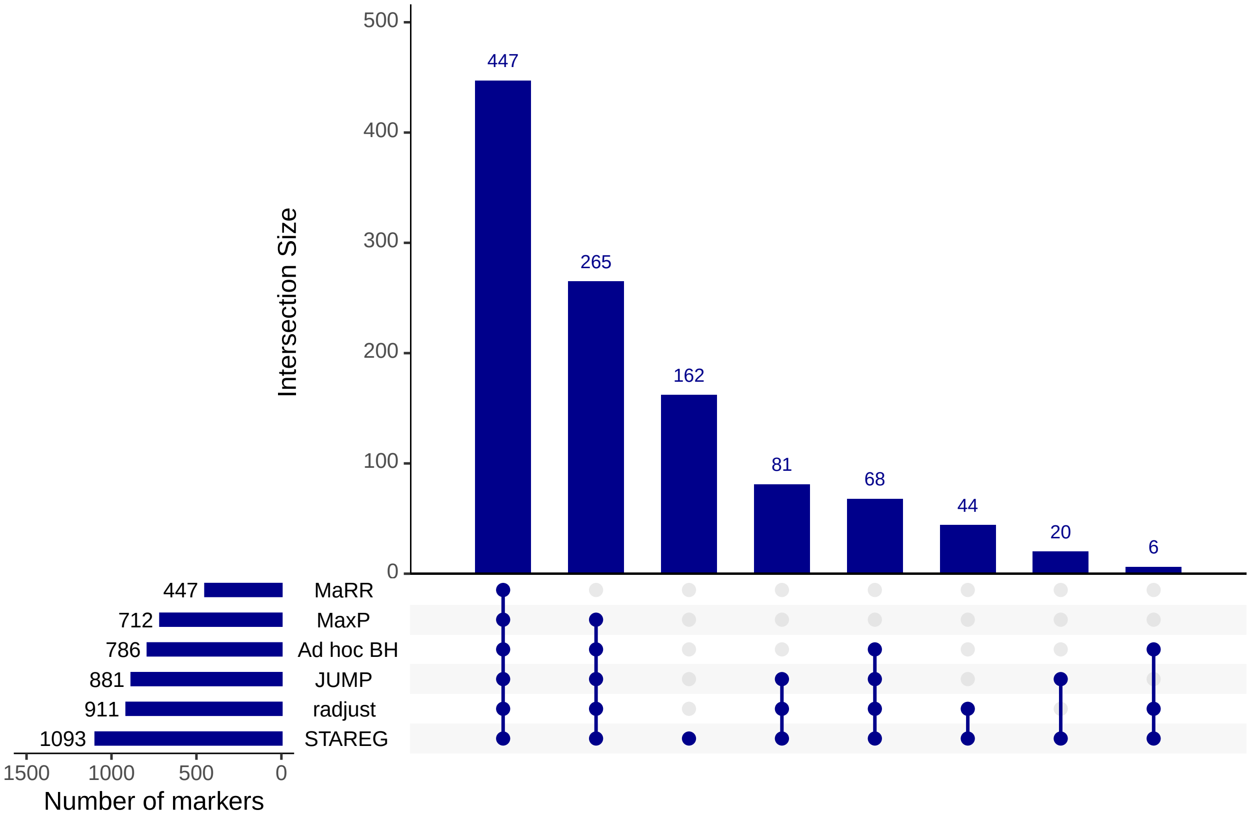


Fig S8: Mouse aortic leukocytes scRNA-seq data analysis results at FDR level 0.01.

enrichment analysis (GSEA) for gene ontology (GO) gene sets [27] with the package R *fgsea* [28]. STAREG ranked genes based on the average of the order statistics of Lfdr. Specifically, let $\widehat{\text{Lfdr}}_{(1)} \leq \ldots \leq \widehat{\text{Lfdr}}_{(m)}$ be the order statistics of $\{\widehat{\text{Lfdr}}_i\}_{i=1}^m$, then genes were ranked based on $\frac{1}{i}\sum_{j=1}^{i}\widehat{\text{Lfdr}}_{(j)}, i = 1, \ldots, m$. In comparison, a common strategy for ranking genes is using $p$-values. Specifically, suppose $p_i$ is the maximum of $p$-values from the two studies for the $i$th gene, and $p_{(1)} \leq \ldots \leq p_{(m)}$ denote the order statistics. Genes were ranked based on $mp_{(i)}/i, i = 1, \ldots, m$. In the GO GSEA of the scRNA data from mouse aortic leukocytes, using Lfdr based ranking, 683 GO terms were enriched at FDR cutoff 0.05, (Fig S9), including all 157 GO terms identified by $p$-value based ranking. The additional identified GO terms include many molecular functions/biological processes/cellular components related to T memory cells and

the immune system, such as leukocyte migration, regulatory T cell differentiation, regulation of antigen receptor-mediated signaling pathway, and immune receptor activity.

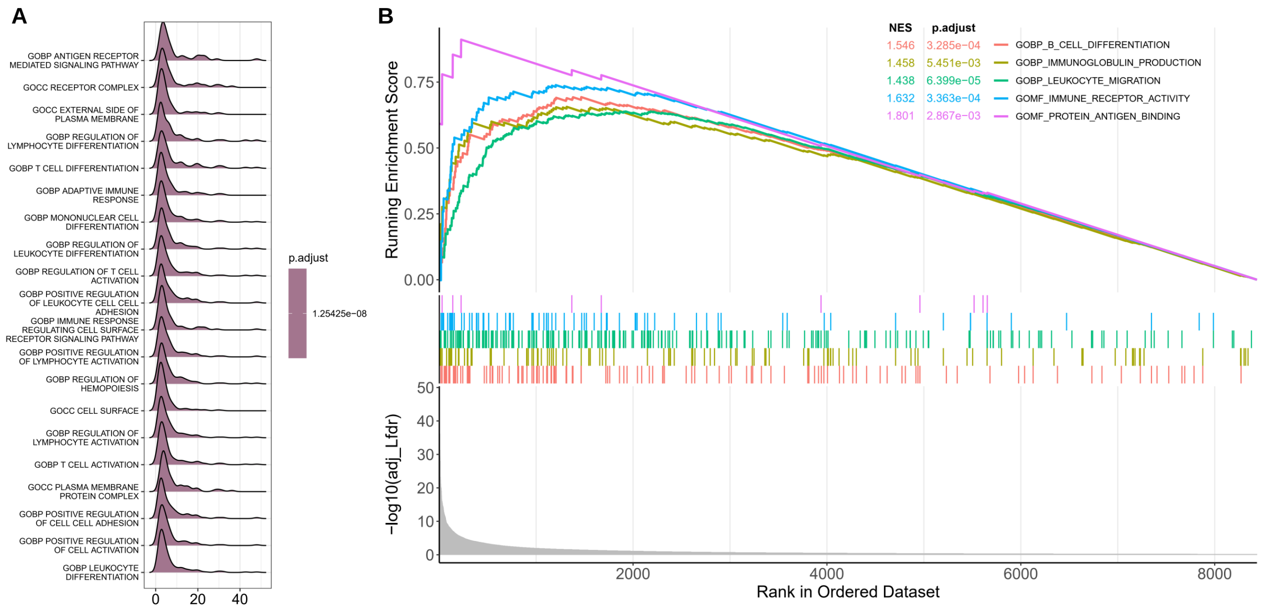


Fig S9: GO GSEA results of the scRNA-seq data from mouse aortic leukocytes, where the ranking is based on $-\log_{10}(\frac{1}{i}\sum_{j=1}^{i}\widehat{\mathrm{Lfdr}}_{(j)})$. (**A**) The ridge plot shows the distribution of genes in the top-20 gene sets enriched by STAREG. (**B**) The running enrichment scores of five representative gene sets only enriched by STAREG at FDR cutoff 0.05.

# E Extending STAREG to more than two studies

## E.1 Problem setup

Suppose we are interested in testing the replicability of $m$ hypotheses in $n$ studies, where a replicable signal means the hypothesis is non-null in all $n$ studies. Let $p_{ji}, j = 1, \ldots, n; i = 1, \ldots, m$ denote the $p$-values of hypothesis $i$ in study $j$. Let $\theta_{ji}$ denote corresponding true hidden states. Assume mixture model for the $p$-values [17, 29]:

$$p_{ji} \mid \theta_{ji} \sim (1-\theta_{ji})f_{j0} + \theta_{ji}f_{j1}, \quad j = 1, \ldots, n; i = 1, \ldots, m,$$

where $f_{j0}, j = 1, \ldots, n$ is the density function of $p$-values under the null, $f_{j1}, j = 1, \ldots, n$ denote the non-null density functions for study $j$. The density functions of $p$-values under the null are assumed to follow standard uniform distribution for all $n$ studies. The heterogeneity across the $n$ studies is accommodated through modeling density functions under the non-null separately by $f_{j1}, j = 1, \ldots, n$. Let $s_i = (\theta_{1i}, \theta_{2i}, \ldots, \theta_{ni}), i = 1, \ldots, m$ denote the joint hidden states across $n$ studies with prior probabilities $P(s_i = (k_1, k_2, \ldots, k_n)) = \xi_{k_1 k_2 \ldots k_n}$, where $k_j \in \{0, 1\}$ for $j = 1, \ldots, n$ and $\sum_{k_1, k_2, \ldots, k_n} \xi_{k_1 k_2 \ldots k_n} = 1$, such that $s_i \in \mathbb{S} = \{0, 1\}^n$. The replicability null hypothesis of $n$ studies is given by

$$H_{0i} : s_i \in \mathbb{H} = \mathbb{S} \backslash \{1\}^n, \quad i = 1, \ldots, m, \tag{S4}$$

where $A \backslash B = \{x \mid x \in A \text{ and } x \notin B\}$. Lfdr is defined as the posterior probability of being null given data. We have

$$\begin{aligned} \text{Lfdr}_i(p_{1i}, p_{2i}, \ldots, p_{ni}) &:= P(s_i \in \mathbb{H} \mid p_{1i}, p_{2i}, \ldots, p_{ni}) \\ &= \frac{\sum_{(k_1, k_2, \ldots, k_n) \in \mathbb{H}} \xi_{k_1 k_2 \ldots k_n} f_{1k_1}(p_{1i}) f_{2k_2}(p_{2i}) \ldots f_{nk_n}(p_{ni})}{\sum_{(k_1, k_2, \ldots, k_n) \in \mathbb{S}} \xi_{k_1 k_2 \ldots k_n} f_{1k_1}(p_{1i}) f_{2k_2}(p_{2i}) \ldots f_{nk_n}(p_{ni})}. \end{aligned} \tag{S5}$$

We impose the monotonicity condition [3, 4]

$$f_{j1}(x)/f_{j0}(x) \text{ are non-increasing in } x, \quad j = 1, \ldots, n.$$

Under the above monotone likelihood condition, $\text{Lfdr}_i$ is monotonically non-decreasing in $(p_{1i}, p_{2i}, \ldots, p_{ni})$ as we have $\text{Lfdr}(x_1^{(1)}, x_2^{(1)}, \ldots, x_n^{(1)}) \leq \text{Lfdr}(x_1^{(2)}, x_2^{(2)}, \ldots, x_n^{(2)})$ for $x_j^{(1)} \leq x_j^{(2)}, j = 1, \ldots, n$. We use the Lfdr defined in (S5) as the test statistic for the replicability null hypothesis specified in (S4).

## E.2 Estimation

To calculate the Lfdr and find the critical value to control the FDR, we estimate the unknown parameters $(\xi_{k_1k_2\ldots k_n})_{(k_1,k_2,\ldots,k_n)\in\mathbb{S}}$ and unknown functions $(f_{j1})_{j=1}^{n}$ by the maximum likelihood estimation.

Assuming conditional independence of the $n$ $p$-value sequences given hidden states, we have

$$f(p_{1i}, p_{2i}, \ldots, p_{ni} \mid \theta_{1i}, \theta_{2i}, \ldots, \theta_{ni}) = \prod_{j=1}^{n} f(p_{ji} \mid \theta_{ji}).$$

Let $\boldsymbol{p}_j = (p_{j1}, p_{j2}, \ldots, p_{jm})$ and $\boldsymbol{\theta}_j = (\theta_{j1}, \theta_{j2}, \ldots, \theta_{jm}), j = 1, \ldots, n.$ The joint log-likelihood function is written by

$$\begin{aligned} l(\boldsymbol{p}_1, \boldsymbol{p}_2, \ldots, \boldsymbol{p}_n, \boldsymbol{\theta}_1, \boldsymbol{\theta}_2, \ldots, \boldsymbol{\theta}_n) = \sum_{i=1}^{m} \Bigg\{ &\sum_{j=1}^{n} \log[(1-\theta_{ji}) f_{j0}(p_{ji}) + \theta_{ji} f_{j1}(p_{ji})] \\ &+ \sum_{(\theta_{1i},\theta_{2i},\ldots,\theta_{ni})\in\{0,1\}^n} (1-\theta_{1i})(1-\theta_{2i})\ldots(1-\theta_{ni}) \log \xi_{\theta_{1i}\theta_{2i}\ldots\theta_{ni}} \Bigg\}. \end{aligned}$$

We use the EM algorithm [1] to maximize the log-likelihood function by iteratively implementing the following two steps.

**E-step**: Given current estimates of $\left(\hat{\xi}^{(t)}_{k_1k_2\ldots k_n}\right)_{(k_1,k_2,\ldots,k_n)\in\mathbb{S}}$ and $\left(f^{(t)}_{j1}\right)_{j=1}^{n}$, calculate the posterior mass probabilities $\gamma^{(t)}_{i,k_1k_2\ldots k_n}$ for $(k_1, k_2, \ldots, k_n) \in \mathbb{S}$ and $i = 1, \ldots, m$ as follows,

$$\begin{aligned} \gamma_{i,k_1k_2\ldots k_n} =& P(s_i = (k_1, k_2, \ldots, k_n) \mid p_{1i}, p_{2i}, \ldots, p_{ni}) \\ =& \frac{\xi_{k_1k_2\ldots k_n} f_{1k_1}(p_{1i}) f_{2k_2}(p_{2i}) \ldots f_{nk_n}(p_{ni})}{\sum_{(k'_1,k'_2,\ldots,k'_n)\in\mathbb{S}} \xi_{k'_1k'_2\ldots k'_n} f_{1k'_1}(p_{1i}) f_{2k'_2}(p_{2i}) \ldots f_{nk'_n}(p_{ni})}. \end{aligned}$$

Define conditional expectation of the log-likelihood function as

$$
\begin{aligned}
&D\left((\xi_{k_1k_2\dots k_n})_{(k_1,k_2,\dots,k_n)\in\mathbb{S}}, (f_{j1})_{j=1}^n \mid \left(\xi^{(t)}_{k_1k_2\dots k_n}\right)_{(k_1,k_2,\dots,k_n)\in\mathbb{S}}, \left(f^{(t)}_{j1}\right)_{j=1}^n\right)\\
=&E_{\boldsymbol{\theta}_1,\boldsymbol{\theta}_2,\dots,\boldsymbol{\theta}_n|(k_1,k_2,\dots,k_n)\in\mathbb{S},\left(f^{(t)}_{j1}\right)_{j=1}^n} l(\boldsymbol{p}_1,\boldsymbol{p}_2,\dots,\boldsymbol{p}_n,\boldsymbol{\theta}_1,\boldsymbol{\theta}_2,\dots,\boldsymbol{\theta}_n)\\
=&\sum_{i=1}^m \sum_{(k_1,k_2,\dots,k_n)\in\mathbb{S}} \gamma^{(t)}_{i,k_1k_2\dots k_n}[\log f_{1k_1}(p_{1i}) + \log f_{2k_2}(p_{2i}) + \cdots + \log f_{nk_n}(p_{ni}) + \log \xi_{k_1k_2\dots k_n}].
\end{aligned}
$$

**M-step**: Update $\left(\hat{\xi}^{(t+1)}_{k_1k_2\dots k_n}\right)_{(k_1,k_2,\dots,k_n)\in\mathbb{S}}$ and $\left(f^{(t+1)}_{j1}\right)_{j=1}^n$ subject to the constraints that $\sum_{(k_1,k_2,\dots,k_n)\in\mathbb{S}} \xi_{k_1k_2\dots k_n} = 1$ and $f_{j1}, j = 1,\dots,n$ are non-increasing density functions. We have

$$
\hat{\xi}^{(t+1)}_{k_1k_2\dots k_n} = \frac{\sum_{i=1}^m \gamma^{(t)}_{i,k_1k_2\dots k_n}}{m},\ (k_1,k_2,\dots,k_n)\in\mathbb{S},
$$

and

$$
\hat{f}^{(t+1)}_{j1} = \arg\max_{\tilde{f}_{j1}\in\mathbb{H}} \left[\sum_{i=1}^m \log \tilde{f}_{j1}(p_{ji}) \sum_{k_j=1;k_1,\dots,k_{j-1},k_{j+1},\dots,k_n\in\{0,1\}} \gamma^{(t)}_{i,k_1k_2\dots k_n}\right],\ j = 1,\dots,n, \quad \text{(S6)}
$$

where $\mathbb{H}$ is the set of p-value density functions under the non-null. We can solve (S6) in exactly the same manner as that in (S1) and (S2).

## E.3 Simulations for three studies

We performed simulations to assess the FDR control and power of STAREG in the replicability analysis of three studies. We generated the joint hidden states from a multinomial distribution with probabilities $P(\theta_{1i} = k, \theta_{2i} = l, \theta_{3i} = r) = \xi_{klr}$ for $k,l,r \in \{0,1\}$. The test statistics for study $j$ ($j = 1,2,3$) were generated from $X_{ji} \mid \theta_{ji} \sim \theta_{ji}N(0,1)+(1-\theta_{ji})N(\mu_j,1), i = 1,\dots,m$, where $\mu_j > 0$. The one-sided $p$-values were generated from $p_{ji} = 1 - \Phi(X_{ji})$ for $i = 1,\dots,m$ and $j = 1,2,3$. As radjust can handle only two studies, we did not include it in the comparison.

We also extend JUMP to three studies, detailed derivations can be found in Section B.3.2.

We set $m = 10,000, \mu_1 = \mu_2 = 2, \xi_{001} = \xi_{010} = \xi_{100}, \xi_{011} = \xi_{101} = \xi_{110} = 0.02$, such that with a specified value of $\xi_{000}$ and $\xi_{001}$, we have $\xi_{111} = 1 - 3\xi_{001} - 3\xi_{011}$. We vary $\mu_3, \xi_{000}, \xi_{001}$ across simulations. For each setting, the FDR and power are calculated based on 100 simulated datasets. We present the FDR control and power comparison at the FDR level 0.05 in Fig S10. The horizontal dashed lines in the FDR plot indicate the target FDR level. We observe that MaRR does not control the FDR. The other methods control the FDR across all simulation settings, where *ad hoc* BH and MaxP are overly conservative. While JUMP has decent power, STAREG has the highest power.

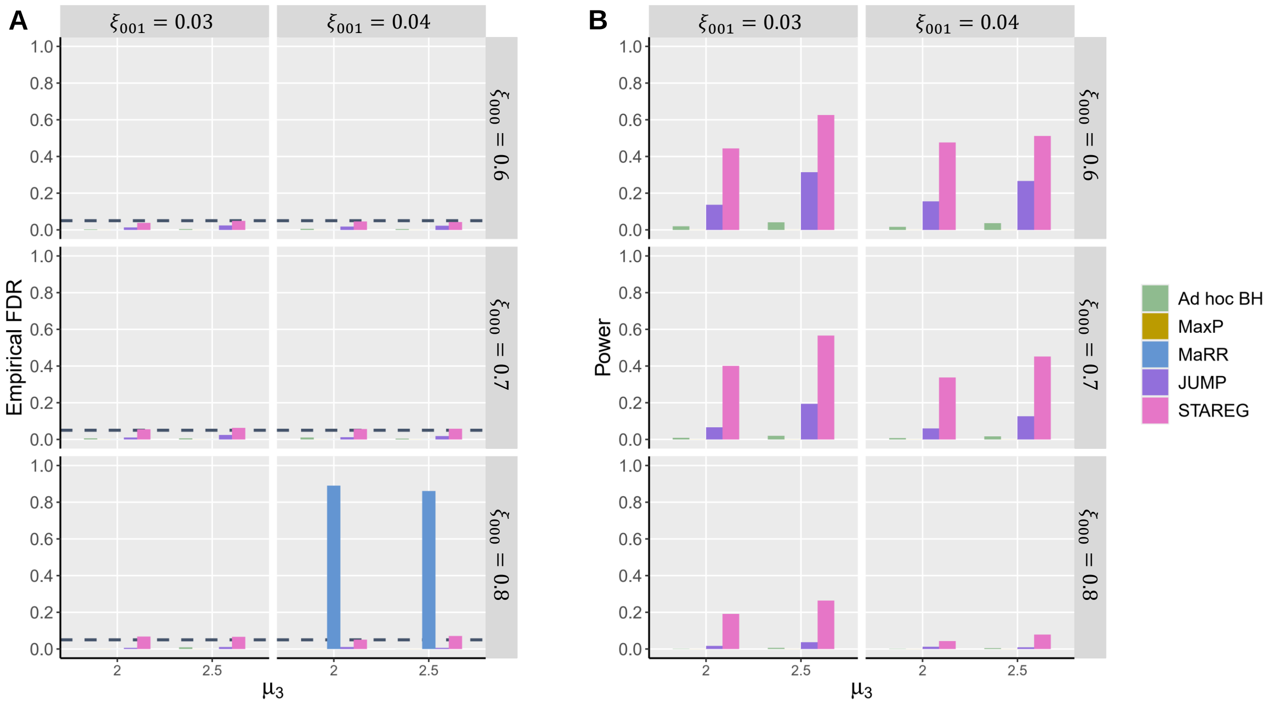


Fig S10: (**A**) FDR control of different methods for three studies. (**B**) Power comparison of different methods for three studies. Simulations were performed with $m = 10,000, \mu_1 = \mu_2 = 2, \xi_{001} = \xi_{010} = \xi_{100}, \xi_{011} = \xi_{101} = \xi_{110} = 0.02$ under different $\mu_3, \xi_{000}$ and $\xi_{001}$. The horizontal dashed line represents the target FDR level of 0.05.

## E.4 Computation time

we examined the run time of different methods when analyzing $m = 10,000$ genes in $n$ studies, where $n = 2, 4, 6, 8$ and 10. We generated the joint hidden states from a multinomial distribution with probabilities $P(\theta_{1i} = k_1, \theta_{2i} = k_2, \ldots, \theta_{ni} = k_n) = \xi_{k_1k_2\ldots k_n}$ for $k_j \in \{0, 1\}, j = 1, 2, \ldots, n$. The test statistics for study $j$ ($j = 1, 2, \ldots, n$) were generated from $X_{ji} \mid \theta_{ji} \sim \theta_{ji}N(0, 1) + (1 - \theta_{ji})N(\mu_j, 1), i = 1, \ldots, m$, where $\mu_j > 0$. The one-sided $p$-values were calculated from $p_{ji} = 1 - \Phi(X_{ji})$ for $i = 1, \ldots, m$ and $j = 1, 2, \ldots, n$. As radjust can handle only two studies and JUMP is not easy to extend, we did not include them in the comparison. We set the $\xi_{k_1k_2\ldots k_n}$ with $k_1 = k_2 = \cdots = k_n = 0$ as 0.6 and the other prior probabilities as $0.4/(n - 1)$, and $\mu_1 = \mu_2 = \cdots = \mu_n = 2$. Table S3 summarizes the computational time of different methods for different values of $n$ in one replication. All methods are implemented in R, in which we use *Rcpp* to speed up the computation. Computations were carried out in an i7-9750H 2.6GHz CPU with 64.0 GB RAM laptop. We observe that STAREG is feasible for 10 studies or so.

Table S3: Computational time (in seconds) for replicability analysis in simulation studies based on normal distributions with different values of $n$.

| Method \ # of studies | $n = 2$ | $n = 4$ | $n = 6$ | $n = 8$ | $n = 10$ |
|---|---|---|---|---|---|
| *Ad hoc* BH | 0.0127 | 0.0164 | 0.0388 | 0.0286 | 0.0384 |
| MaxP | 0.0235 | 0.0221 | 0.0234 | 0.0275 | 0.0350 |
| MaRR | 2.2867 | 1.7575 | 1.6570 | 2.1905 | 2.2003 |
| STAREG | 0.0364 | 0.0641 | 0.1179 | 6.9681 | 26.5137 |

# References


[1] Arthur P Dempster, Nan M Laird, and Donald B Rubin. Maximum likelihood from incomplete data via the em algorithm. *Journal of the Royal Statistical Society: Series B (Methodological)*, 39(1):1–22, 1977.

[2] Tim Robertson, Richard L. Dykstra, and F. T. Wright. Order restricted statistical inference. In *Wiley Series in Probability and Mathematical Statistics*. John Wiley and Sons, 1988.

[3] Wenguang Sun and T Tony Cai. Oracle and adaptive compound decision rules for false discovery rate control. *Journal of the American Statistical Association*, 102(479):901–912, 2007.

[4] Hongyuan Cao, Wenguang Sun, and Michael R Kosorok. The optimal power puzzle: scrutiny of the monotone likelihood ratio assumption in multiple testing. *Biometrika*, 100(2):495–502, 2013.

[5] Hongyuan Cao, Jun Chen, and Xianyang Zhang. Optimal false discovery rate control for large scale multiple testing with auxiliary information. *Annals of Statistics*, 50(2):807, 2022.

[6] Richard E Barlow and Hugh D Brunk. The isotonic regression problem and its dual. *Journal of the American Statistical Association*, 67(337):140–147, 1972.

[7] Yoav Benjamini, Ruth Heller, and Daniel Yekutieli. Selective inference in complex research. *Philosophical Transactions of the Royal Society A: Mathematical, Physical and Engineering Sciences*, 367(1906):4255–4271, 2009.

[8] Kenneth Hung and William Fithian. Rank verification for exponential families. *The Annals of Statistics*, 47(2):758 – 782, 2019.

[9] Daisy Philtron, Yafei Lyu, Qunhua Li, and Debashis Ghosh. Maximum rank reproducibility: a nonparametric approach to assessing reproducibility in replicate experiments. *Journal of the American Statistical Association*, 113(523):1028–1039, 2018.

[10] Marina Bogomolov and Ruth Heller. Assessing replicability of findings across two studies of multiple features. *Biometrika*, 105(3):505–516, 2018.

[11] Pengfei Lyu, Yan Li, Xiaoquan Wen, and Hongyuan Cao. Jump: replicability analysis of high-throughput experiments with applications to spatial transcriptomic studies. *Bioinformatics*, 39(6):btad366, 2023.

[12] Yoav Benjamini and Yosef Hochberg. Controlling the false discovery rate: a practical and powerful approach to multiple testing. *Journal of the Royal Statistical Society: Series B (Methodological)*, 57(1):289–300, 1995.

[13] John D Storey. A direct approach to false discovery rates. *Journal of the Royal Statistical Society: Series B (Statistical Methodology)*, 64(3):479–498, 2002.

[14] John D Storey, Jonathan E Taylor, and David Siegmund. Strong control, conservative point estimation and simultaneous conservative consistency of false discovery rates: a unified approach. *Journal of the Royal Statistical Society: Series B (Statistical Methodology)*, 66(1):187–205, 2004.

[15] John D Storey and Robert Tibshirani. Statistical significance for genomewide studies. *Proceedings of the National Academy of Sciences*, 100(16):9440–9445, 2003.

[16] Shiquan Sun, Jiaqiang Zhu, and Xiang Zhou. Statistical analysis of spatial expression patterns for spatially resolved transcriptomic studies. *Nature Methods*, 17(2):193–200, 2020.

[17] Bradley Efron, Robert Tibshirani, John D Storey, and Virginia Tusher. Empirical bayes analysis of a microarray experiment. *Journal of the American Statistical Association*, 96(456):1151–1160, 2001.

[18] Patrik L Ståhl, Fredrik Salmén, Sanja Vickovic, Anna Lundmark, José Fernández Navarro, Jens Magnusson, Stefania Giacomello, Michaela Asp, Jakub O Westholm, Mikael Huss, et al. Visualization and analysis of gene expression in tissue sections by spatial transcriptomics. *Science*, 353(6294):78–82, 2016.

[19] Jiaqiang Zhu, Shiquan Sun, and Xiang Zhou. SPARK-X: non-parametric modeling enables scalable and robust detection of spatial expression patterns for large spatial transcriptomic studies. *Genome Biology*, 22(1):1–25, 2021.

[20] Patrick AP Moran. Notes on continuous stochastic phenomena. *Biometrika*, 37(1/2):17–23, 1950.

[21] Oscar Franzén, Li-Ming Gan, and Johan L M Björkegren. PanglaoDB: a web server for exploration of mouse and human single-cell rna sequencing data. *Database*, 2019:baz046, 2019.

[22] Samarendra Das, Anil Rai, Michael L Merchant, Matthew C Cave, and Shesh N Rai. A comprehensive survey of statistical approaches for differential expression analysis in single-cell rna sequencing studies. *Genes*, 12(12):1947, 2021.

[23] Jeanette Ampudia, William W Young-Greenwald, Jana Badrani, Sole Gatto, Adam Pavlicek, Taylor Doherty, Stephen Connelly, and Cherie T Ng. CD6-ALCAM signaling regulates multiple effector/memory T cell functions. *The Journal of Immunology*, 204(1_Supplement):150–13, 2020.

[24] Socheatraksmey Ung, Pongsakorn Choochuen, Wannakorn Khopanlert, Kajornkiat Maneechai, Surasak Sangkhathat, Seitaro Terakura, and Jakrawadee Julamanee. Enrich-

ment of T-cell proliferation and memory gene signatures of CD79A/CD40 costimulatory domain potentiates CD19CAR-T cell functions. *Frontiers in Immunology*, 13, 2022.

[25] Emily Corse, Rachel A Gottschalk, and James P Allison. Strength of TCR–peptide/MHC interactions and in vivo T cell responses. *The Journal of Immunology*, 186(9):5039–5045, 2011.

[26] Mark A Daniels and Emma Teixeiro. TCR signaling in T cell memory. *Frontiers in Immunology*, 6:617, 2015.

[27] Suzi A Aleksander, James Balhoff, Seth Carbon, J Michael Cherry, Harold J Drabkin, Dustin Ebert, Marc Feuermann, Pascale Gaudet, Nomi L Harris, et al. The gene ontology knowledgebase in 2023. *Genetics*, 224(1):iyad031, 2023.

[28] Gennady Korotkevich, Vladimir Sukhov, Nikolay Budin, Boris Shpak, Maxim N. Artyomov, and Alexey Sergushichev. Fast gene set enrichment analysis. *bioRxiv*, 2021.

[29] Bradley Efron. *Large-scale inference: empirical Bayes methods for estimation, testing, and prediction*, volume 1. Cambridge University Press, 2012.
