# Supplementary material for: STAREG: Statistical replicability analysis of high throughput experiments with applications to spatial transcriptomic studies
Source: PLoS Genet. 2024 Oct 3;20(10):e1011423. doi: 10.1371/journal.pgen.1011423 (PMC11478871; doi:10.1371/journal.pgen.1011423)
Supplement: Attachment — Submitted filename: Response_letter.pdf [file pgen.1011423.s006.pdf]

Dear Editor,

A revised version of our paper (PGENETICS-D-24-00265) and our response to the referee's comments are attached. We want to thank you and the referees for the very detailed and helpful comments that led to an improved revision.

Yours sincerely,

Li, Y., Zhou, X., Chen, Rui, Zhang, X. and Cao, H.

Response to #1 Reviewer's Comments

General comments. *In this paper, the authors present a new method for detecting replicable signals across two related studies, and illustrate its application to replicable SVG detection. The method is empirical Bayes-based, aims to jointly analyze a pair of p-values. The method seems to be statistical rigorous and computational scalable. Detect replicable biological findings is of interest and method definitely has merits. However, I have the following questions.*

1. *I found it rather puzzling that the authors seem to name their method STAREG in the Introduction section and mentioned a few times. But this name completely disappeared in Results, Methods and discussion sections. In all the figures, the authors used "our method" as the label. This is very strange and confusing. Please be consistent.*

Response: We revised the manuscript to make the pronouns of the proposed method consistent in figures and texts.

2. *Lines 102-103, "Suppose we have two datasets obtained by measuring the spatial expression patterns of m genes under distinct cellular environments with different technologies". This is problematic. By distinct, are authors referring to two different tissues, or under different conditions? If so, then it is expected that a gene may be SVG in one experiment, but non-SVG is the other. Since it is highly likely that a gene being SVG is context-dependent. I would be more comfortable if the two experiments are highly-related.*

Response: We apologize for not presenting it clearly. We focus on replicability analysis of two independent yet conceptually highly related samples. For instance, when analyzing olfactory bulb data from two mice, a true SVG should exhibit spatial patterns in both mice though the spatial patterns and tissue sections can be different (Figure 5, Supplementary Figure S6, S7 and S12). Please see lines 78-82 on page 4 of the main paper.

3. *Figure 2. It seems that line style changed from a, b to c? Why not consistent?*

Response: We fixed it. Please see Figure R1 and Figure R2 below. You can find them in the main paper as Figure 2 and Figure 3 on pages 8-9.

4. *I was very surprised to see that the numbers of genes and spots do not change at all after filtering. Are you sure? If so, what is the point of filtering?*

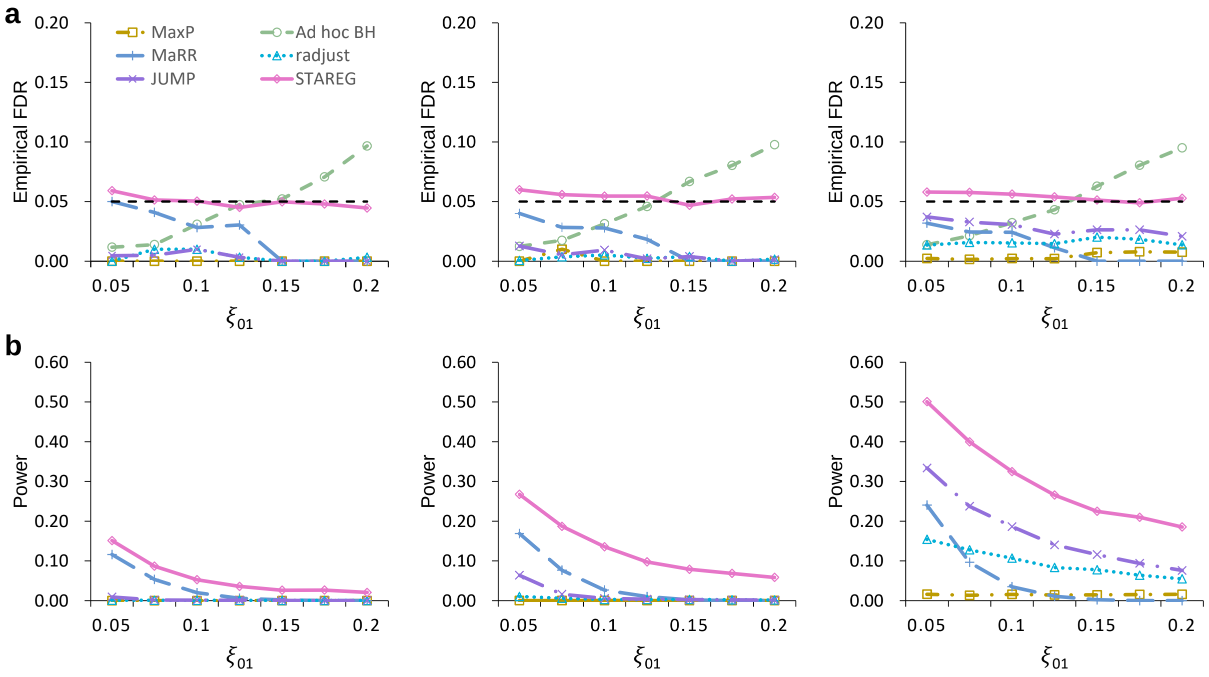

Figure R1: Performance comparisons of different methods in simulation studies based on normal distributions with different ξ_{01}. Left: $\mu_1 = \mu_2 = 2, \sigma_1 = \sigma_2 = 1$. Middle: $\mu_1 = 2, \mu_2 = 2.5, \sigma_1 = \sigma_2 = 1$. Right: $\mu_1 = \mu_2 = 2, \sigma_1 = 1, \sigma_2 = 0.5$. All simulations were conducted under the setting of $m = 10,000$, $\xi_{11} = 0.05$ and $\xi_{01} = \xi_{10}$. The target FDR level is 0.05 represented by horizontal dashed lines in (**a**), and the results were calculated over 100 runs. We do not present the power of *ad hoc* BH in (**b**) as it fails to control FDR.

Response: Thank you for the careful reading. We apologize for presenting incorrect information on the original ST data sets for the mouse olfactory bulb (MOB). The Replicate 1 data (file "MOB Replicate 1") consist of 16,573 genes on 265 spots, and the Replicate 8 data (file "MOB Replicate 8") contain 15,288 genes on 234 spots. After filtering out genes that are expressed in less than 10% of the spatial locations and selecting spatial locations with at least ten total read counts, we obtain 10,373 genes on 265 spots in the Replicate 1 dataset and 9,671 genes on 232 spots in the Replicate 8 dataset. Please see main paper page 12 lines 200-206.

5.*Line 177-179. I wonder how many genes identified by other methods as SVG, but not by STAREG?*

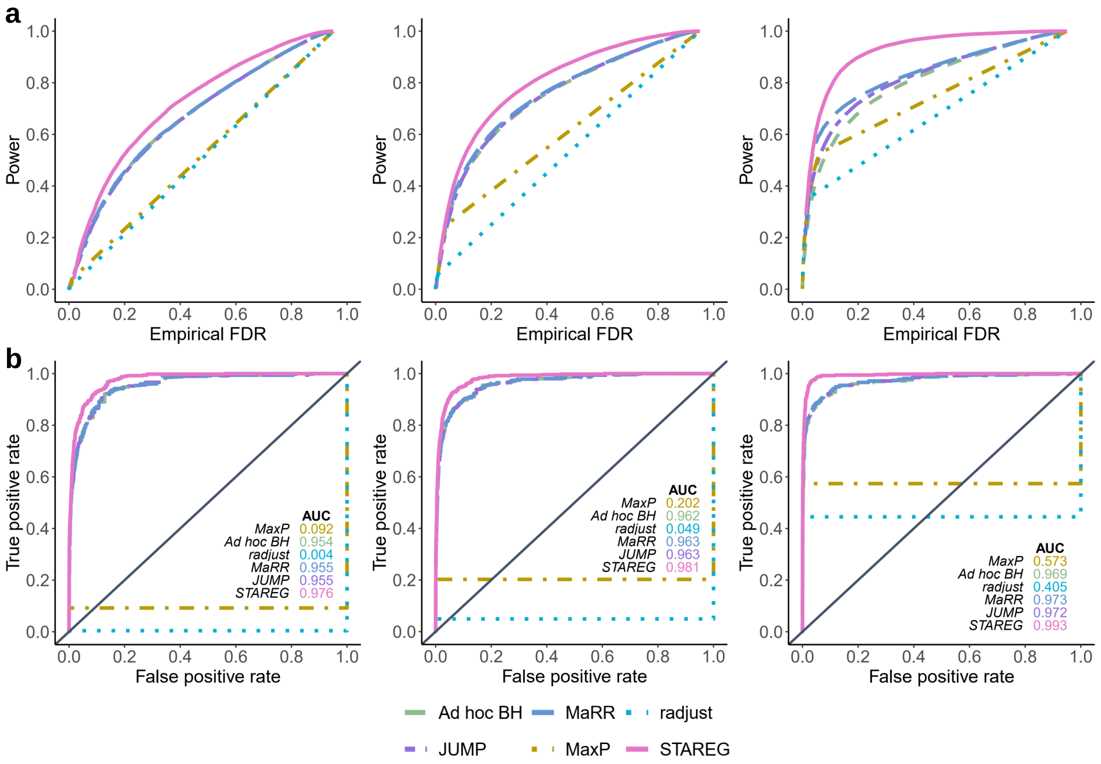

Figure R2: Performance comparisons of different methods in simulation studies based on normal distributions with different levels of nominal FDR. Left: $\mu_1 = \mu_2 = 2, \sigma_1 = \sigma_2 = 1$. Middle: $\mu_1 = 2, \mu_2 = 2.5, \sigma_1 = \sigma_2 = 1$. Right: $\mu_1 = \mu_2 = 2, \sigma_1 = 1, \sigma_2 = 0.5$. All simulations were performed under the setting of $m = 10,000, \xi_{00} = 0.9, \xi_{01} = \xi_{10} = 0.025$ and $\xi_{11} = 0.05$. The Receiver Operating Characteristic (ROC) curves and corresponding Area Under Curve (AUC) values in (**b**) were obtained from one replication. The diagonal dashed line with slope 1 is used as a reference.

Response: Figure 3b and Figure 5b in the revised manuscript show the number of SVGs identified by different methods. For the ST data from MOB (Figure 5(b)), we observe that MaRR detected 14 replicable SVGs, which were not identified by STAREG, and radjust detected 1 replicable SVG, which was not identified by STAREG. For the Slide-seq and Slide-seqV2 data from mouse cerebellum (MC) (Figure 7(b)), all replicable SVGs identified by competing methods were also detected by STAREG.

Response to #2 Reviewer's Comments

General Comments. *Li et al. introduces a statistical method for identifying replicable differential genes. While the method aims to address the issue of replicability across studies and demonstrates solidity, the rationale for focusing on spatial transcriptomics data lacks clear justification. Moreover, additional real data applications are in need to strengthen the manuscript's support for the method's accuracy.*

Response: This is an excellent question. Our method is applicable to replicability analysis in general. As an exciting new technology, no existing work looks at the replicability issue of spatial transcriptomic data, and this motivates us to have such a focus in the paper. To strengthen the manuscript, we have added results from the single cell data analysis. Please see Supplementary Section D.2.

1. *I wonder why the authors choose spatial transcriptomics as the application to demonstrate the performance of this method. Even when a gene is spatially variable in two studies, its spatial expression patterns can be quite different, so such a test may not be of strong interest in real practice. A more useful application would be single-cell gene expression analysis, where one tries to determine if a gene is up- or down-regulated in both studies.*

Response: Analyzing single-cell gene expression data is an excellent suggestion. We downloaded scRNA-seq data from two mouse aortic leukocyte samples measured with 10x chromium from PanglaoDB [Franzén et al., 2019] (`https://panglaodb.se/samples.html`). We treat sample SRS2747908 as study 1, which includes read counts of $19,150$ genes in $1,629$ cells, and sample SRS2747908 as study 2, which includes count data of $21,052$ genes in $1,608$ cells. After filtering out genes expressed in less than 10 cells and selecting cells with at least $1,000$ total read counts, we obtained $10,512$ genes in 398 cells in study 1 and $12,868$ genes in $1,090$ cells in study 2. We used the cluster results published in PanglaoDB [Franzén et al., 2019], resulting in 8 clusters with more than 10 cells in study 1 and 17 clusters with more than 10 cells in study 2, respectively. We further filtered out cells not in any of the above clusters, yielding 378 cells in Study 1 and $1,036$ cells in Study 2. We performed a differential analysis of T memory cells with the remaining cells using the Wilcox test [Das et al., 2021], and got p-values. Next, we took common genes in both studies and obtained $10,274$ pairs of p-values as input for subsequent replicability analysis.

The results of different methods at the FDR level 0.01 are summarized in Figure R3. MaRR identified 447 replicable markers for T memory cells, MaxP detected an additional 265, and these 712 replicable markers were also detected by all other methods. *Ad hoc* BH identified 786 replicable markers, JUMP identified 881 replicable markers, and radjust identified 911 replicable markers. STAREG detected $1,093$ replicable markers, including all those identified by other methods, and an additional 162 not detected by any other method.

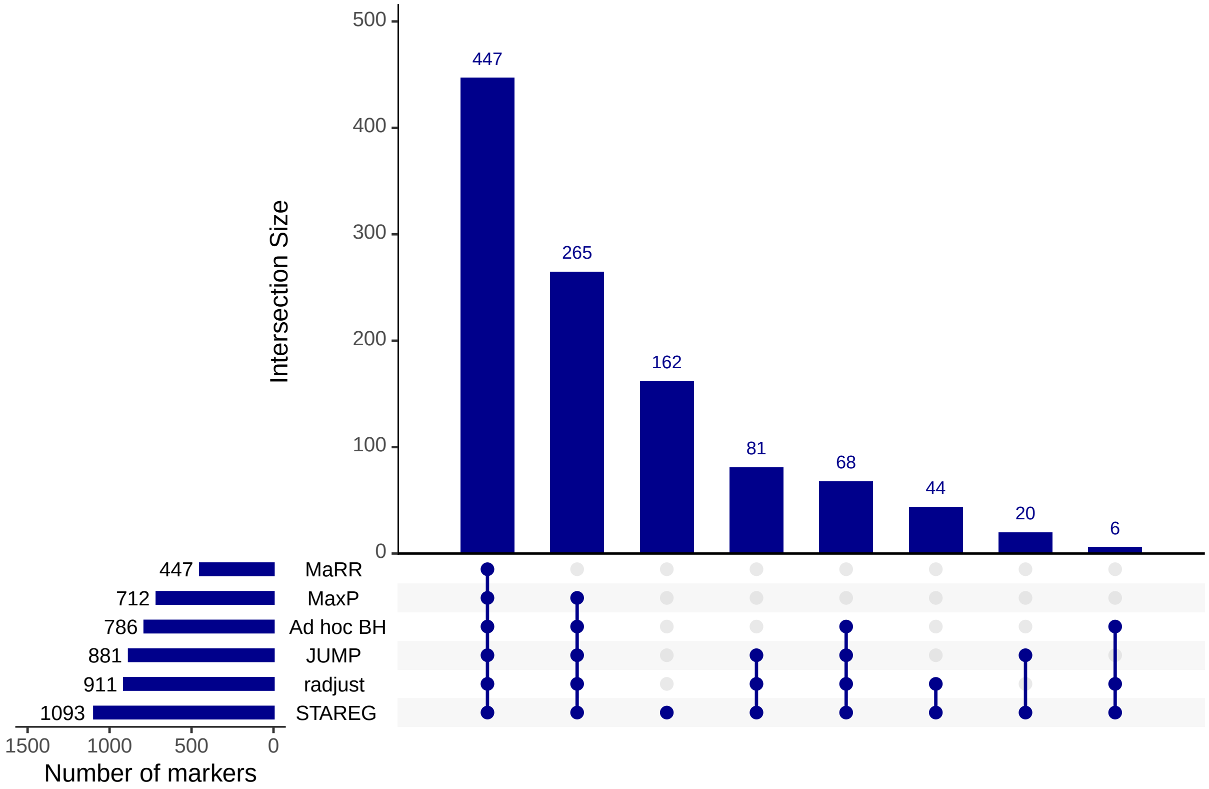

Figure R3: Mouse aortic leukocytes scRNA-seq data analysis results at FDR level 0.01.

To examine the biological relevance of replicable markers, we performed a gene set enrichment analysis (GSEA) for gene ontology (GO) gene sets [Aleksander et al., 2023] with the package R *fgsea* [Korotkevich et al., 2021]. STAREG ranked genes based on the average of the order statistics of Lfdr. Specifically, let $\widehat{\text{Lfdr}}_{(1)} \leq \ldots \leq \widehat{\text{Lfdr}}_{(m)}$ be the order statistics of $\{\widehat{\text{Lfdr}}_i\}_{i=1}^m$, then genes were ranked based on $\frac{1}{i}\sum_{j=1}^i \widehat{\text{Lfdr}}_{(j)}, i = 1, \ldots, m$. In comparison, a common strategy for ranking genes is using p-values. Specifically, suppose

p_i is the maximum of p-values from the two studies for the ith gene, and $p_{(1)} \leq \ldots \leq p_{(m)}$ denote the order statistics. Genes were ranked based on $mp_{(i)}/i, i = 1, \ldots, m$.

In the GO GSEA of the scRNA data from mouse aortic leukocytes, using Lfdr based ranking, 683 GO terms were enriched at FDR cutoff 0.05, (Figure R4), including all 157 GO terms identified by p-value based ranking. The additional identified GO terms include many molecular functions/biological processes/cellular components related to T memory cells and the immune system, such as leukocyte migration, regulatory T cell differentiation, regulation of antigen receptor-mediated signaling pathway, and immune receptor activity.

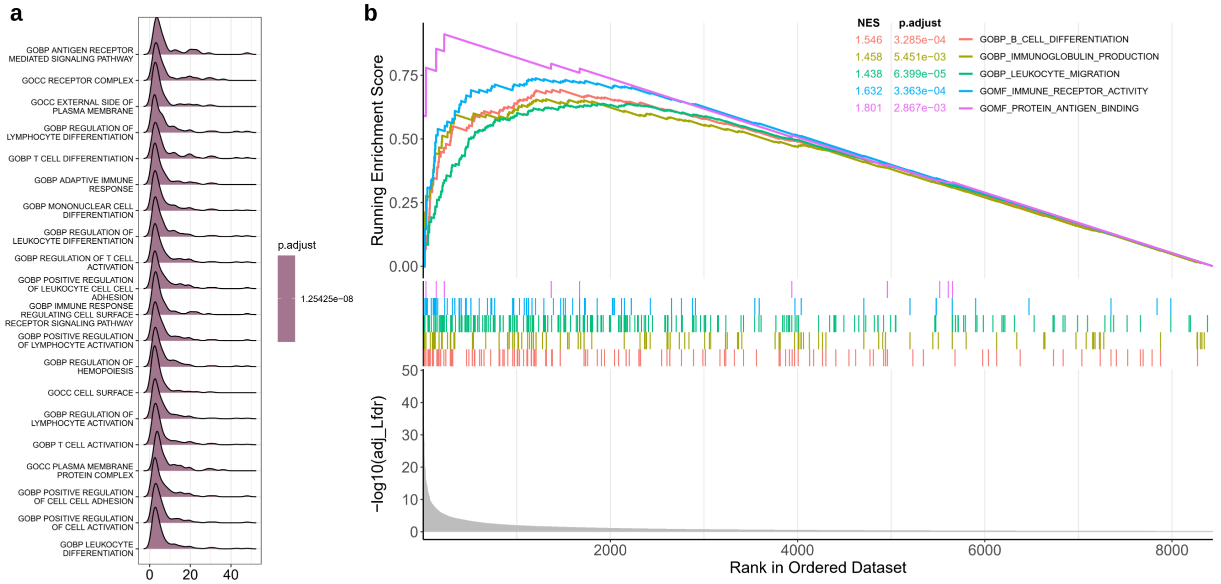

Figure R4: GO GSEA results of the scRNA-seq data from mouse aortic leukocytes, where the ranking is based on $-\log_{10}(\frac{1}{i}\sum_{j=1}^{i}\widehat{\mathrm{Lfdr}}_{(j)})$. (**a**) The ridge plot shows the distribution of genes in the top-20 gene sets enriched by STAREG. (**b**) The running enrichment scores of five representative gene sets only enriched by STAREG at FDR cutoff 0.05.

We added the above results in Supplementary Section D.2 on pages 19-20.

2. *The current method considers the scenario with two studies. However, many joint single-cell analyses involve more than two studies/datasets. How easy is it to extend the method for a more general scenario?*

Response: In theory, it is straightforward to extend the current method to more than two studies since we only need to add additional components to the mixture model.

The derivation can be found in the Supplementary E.1 and E.2. In practice, this can be computationally expensive if the number of studies is large, as the computational time scales exponentially. In the following, we present simulation results for three studies.

We generated the joint hidden states from a multinomial distribution with probabilities $P(\theta_{1i} = k, \theta_{2i} = l, \theta_{3i} = r) = \xi_{klr}$ for $k, l, r \in \{0, 1\}$. The test statistics for study j ($j = 1, 2, 3$) were generated from $X_{ji} \mid \theta_{ji} \sim \theta_{ji} N(0, 1) + (1 - \theta_{ji}) N(\mu_j, 1), i = 1, \ldots, m$, where $\mu_j > 0$. The one-sided p-values were generated from $p_{ji} = 1 - \Phi(X_{ji})$ for $i = 1, \ldots, m$ and $j = 1, 2, 3$. As radjust can handle only two studies, we did not include it in the comparison. We also extend JUMP to three studies, detailed derivations can be found in the Supplementary Material. We set $m = 10,000, \mu_1 = \mu_2 = 2, \xi_{001} = \xi_{010} = \xi_{100}, \xi_{011} = \xi_{101} = \xi_{110} = 0.02$, such that with a specified value of ξ_{000} and ξ_{001}, we have $\xi_{111} = 1 - 3\xi_{001} - 3\xi_{011}$. We vary $\mu_3, \xi_{000}, \xi_{001}$ across simulations. For each setting, the FDR

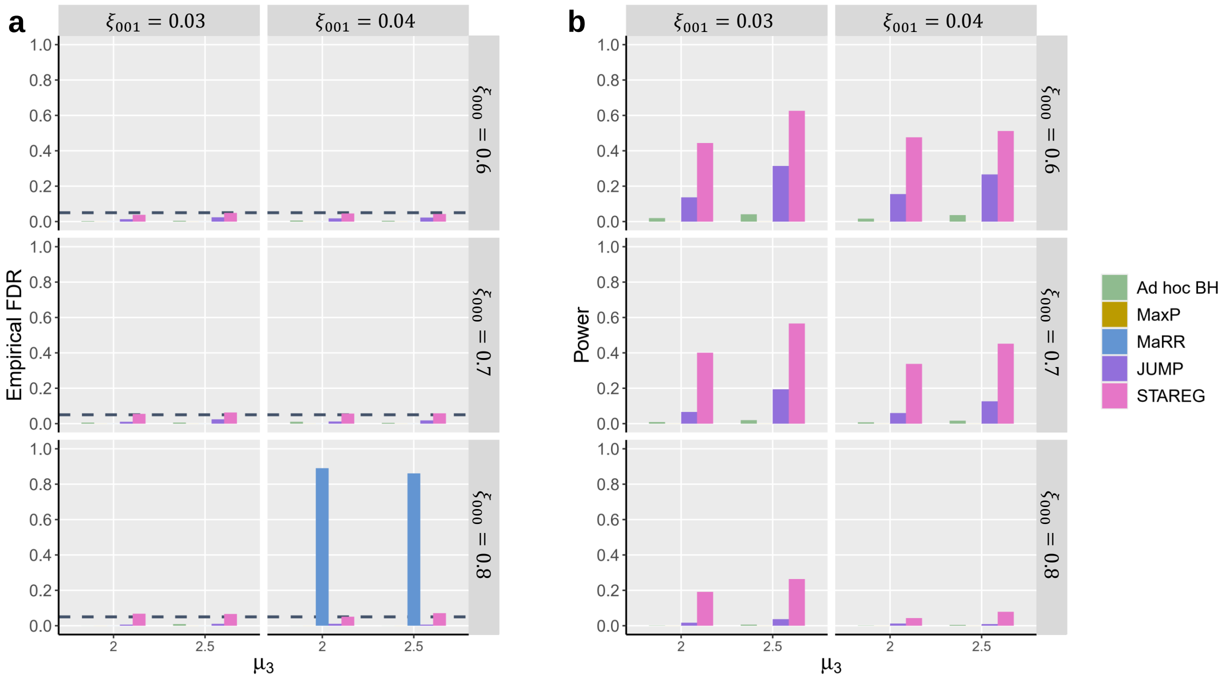

Figure R5: (**a**) FDR control of different methods for three studies. (**b**) Power comparison of different methods for three studies. Simulations were performed with $m = 10,000, \mu_1 = \mu_2 = 2, \xi_{001} = \xi_{010} = \xi_{100}, \xi_{011} = \xi_{101} = \xi_{110} = 0.02$ under different μ_3, ξ_{000} and ξ_{001}. The horizontal dashed line represents the target FDR level of 0.05.

and power are calculated based on 100 runs. We present the FDR control and power comparison at the FDR level 0.05 in Figure R5. The horizontal dashed lines in the FDR plot indicate the target FDR level. We observe that MaRR does not control the FDR. The other methods control the FDR across all simulation settings, where *ad hoc* BH and MaxP are overly conservative. While JUMP has decent power, STAREG has the highest power.

Moreover, we examined the run time of different methods when analyzing $m = 10,000$ genes in n studies, where $n = 2, 4, 6, 8$ and 10. We generated the joint hidden states from a multinomial distribution with probabilities $P(\theta_{1i} = k_1, \theta_{2i} = k_2, \ldots, \theta_{ni} = k_n) = \xi_{k_1 k_2 \ldots k_n}$ for $k_j \in \{0, 1\}, j = 1, 2, \ldots, n$. The test statistics for study j ($j = 1, 2, \ldots, n$) were generated from $X_{ji} \mid \theta_{ji} \sim \theta_{ji} N(0, 1) + (1 - \theta_{ji}) N(\mu_j, 1), i = 1, \ldots, m$, where $\mu_j > 0$. The one-sided p-values were calculated from $p_{ji} = 1 - \Phi(X_{ji})$ for $i = 1, \ldots, m$ and $j = 1, 2, \ldots, n$. As radjust can handle only two studies and JUMP is not easy to extend, we did not include them in the comparison. We set the $\xi_{k_1 k_2 \ldots k_n}$ with $k_1 = k_2 = \cdots = k_n = 0$ as 0.6 and the other prior probabilities as $0.4/(n-1)$, and $\mu_1 = \mu_2 = \cdots = \mu_n = 2$. Table R1 summarizes the computational time of different methods for different values of n in one replication. All methods are implemented in R, in which we use *Rcpp* to speed up the computation. Computations were carried out in an Intel(R) Core(TM) i7-9750H 2.6GHz CPU with 64.0 GB RAM laptop. We observe that STAREG is feasible for 10 studies or so.

Table R1: Computational time (in seconds) of analyzing n studies with different methods.

Method \ # of studies	$n = 2$	$n = 4$	$n = 6$	$n = 8$	$n = 10$
Ad hoc BH	0.0127	0.0164	0.0388	0.0286	0.0384
MaxP	0.0235	0.0221	0.0234	0.0275	0.0350
MaRR	2.2867	1.7575	1.6570	2.1905	2.2003
STAREG	0.0364	0.0641	0.1179	6.9681	26.5137

We added the above results in Supplementary Section E.3 and E.4 on pages 23-24. Please see the main paper page 21 lines 351-357.

3. *The manuscript focuses on single-cell analysis as the application, but the simulation*

is based on simple normal distributions. There have been many single-cell data simulators available. What if the authors used some of these tools to generate data that mimic real single-cell data characteristics?

Response: Our main application is spatial transcriptomic data, not single-cell data. To this end, we performed realistic simulations where spatial transcriptomic data are generated from SRTsim [Zhu et al., 2023] in addition to realistic simulations based on parameters inferred from MOB data [Ståhl et al., 2016] in the original manuscript. SRTsim maintains various expression characteristics of SRT data and preserves spatial patterns. In each simulation, we used pre-specified $\xi_{00}, \xi_{01}, \xi_{10}$ and ξ_{11} to generate hidden states (θ_1, θ_2) where $P(\theta_1 = k, \theta_2 = l) = \xi_{kl}, k, l = 0, 1$ for m genes from multinomial distribution. We then separately generated gene expression count data for two studies using SRTsim based on simulated θ_1 and θ_2. We generated 384 and 486 spatial location coordinates based on circular tissue shapes by random sampling for the two studies, respectively. Gene count data were generated by the zero-inflated Poisson model embedded in SRTsim. The dispersion, zero-proportion, and mean parameters were set to 0.2, 0.01, and 2, respectively. The spatial spots for each study were divided into two spatial domains. For non-SVGs, the fold change is set to 1 across all spots; for SVGs, the fold change is set to 1 in the red domain and 3 in the blue domain as in Figure R6. After obtaining the gene expression count matrices for two studies, we applied SPARK-X [Zhu et al., 2021] to produce paired p-values as inputs of the replicability analysis.

We set $m = 10,000$, $\xi_{11} = 0.05$ and $\xi_{01} = \xi_{10}$ across all simulations, such that with a specified ξ_{00}, we have $\xi_{01} = \xi_{10} = (1 - \xi_{00} - \xi_{11})/2$. We varied $\xi_{00} \in \{0.65, 0.75, 0.85\}$ to evaluate the performance of different methods. For each setting, the empirical FDR and power of different methods are calculated based on 100 simulated datasets. Figure R6 shows the empirical FDR and power with nominal FDR levels ranging from 0.01 to 0.2. We observe that *ad hoc* BH fails to control the FDR when the two studies are relatively heterogeneous (e.g., $\xi_{00} = 0.65$), whereas MaRR was overly conservative. MaxP was conservative across all settings. JUMP and radjust showed decent power while maintaining FDR control. STAREG properly controlled FDR across all settings and had the highest power at the same nominal FDR levels.

We added these in the main paper Section 2.2.2 on pages 10-12.

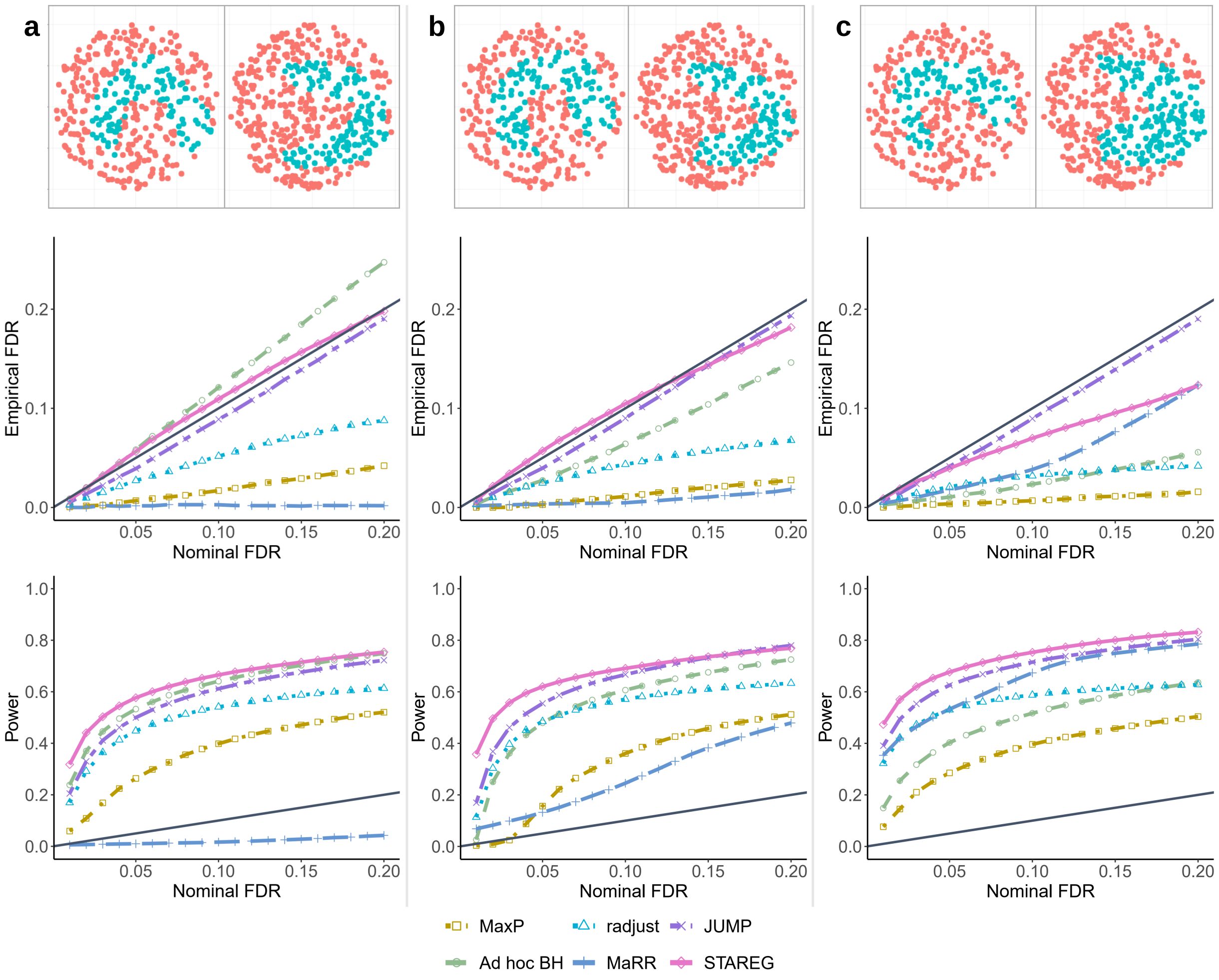

Figure R6: Empirical FDR and power of different methods in realistic simulations based on SRTsim [Zhu et al., 2023]. Each column corresponds to a different value of ξ_{00} (**a**: $\xi_{00} = 0.65$; **b**: $\xi_{00} = 0.75$; **c**: $\xi_{00} = 0.85$.) Simulations were conducted under the setting of $m = 10,000$, $\xi_{11} = 0.05$ and $\xi_{01} = \xi_{10}$. The nominal FDR level ranges from 0.01 to 0.2, and the results were calculated over 100 replications.

4. *An important assumption of the proposed method is that the p-values are uniformly distributed under the null. We know that this is often more or less violated when a method is applied to real data. Therefore, in addition to SPARK, it would be helpful to apply additional SVG methods to real data, and evaluate how the proposed methods perform given p-values produced by different methods. How much would the results differ between each other?*

Response: Currently, we use SPARK [Sun et al., 2020] to analyze the MOB data.

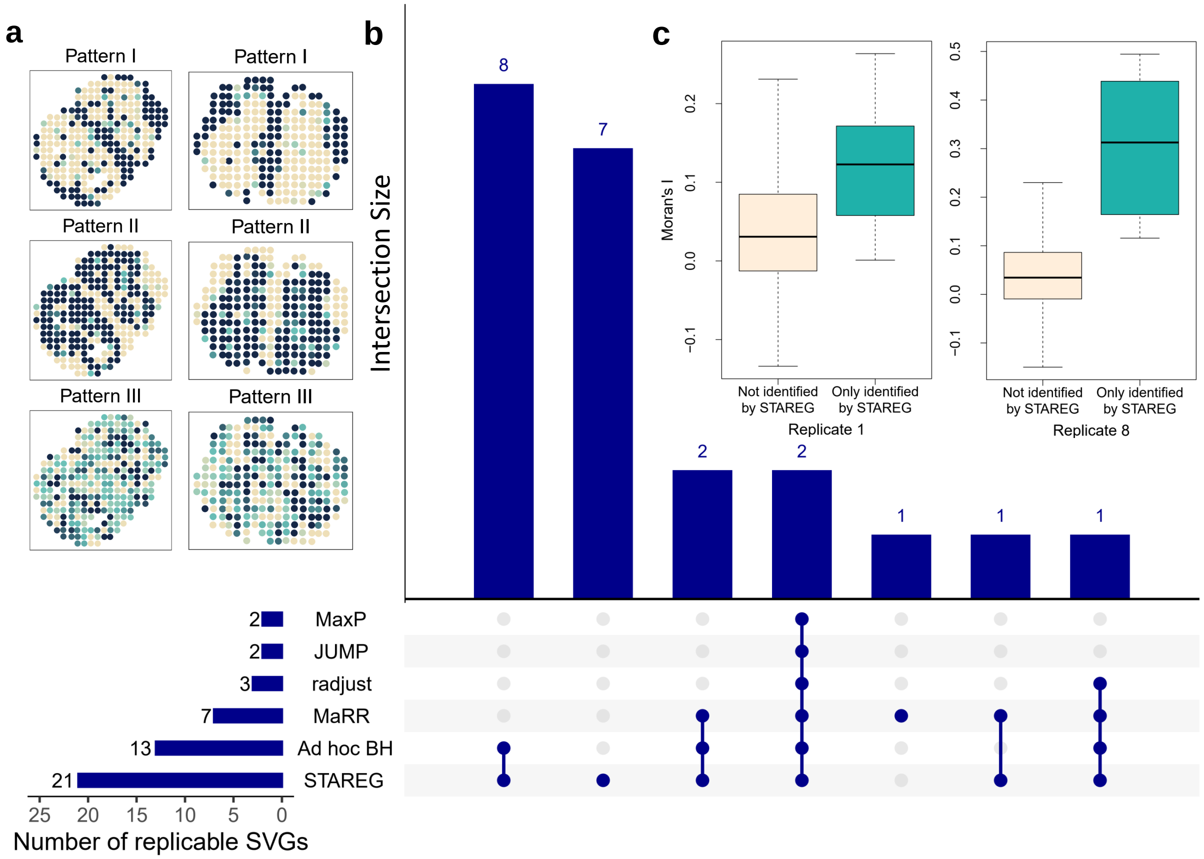

Figure R7: MOB data analysis results with SPARK-X at FDR level 0.05. (**a**) Three spatial patterns summarized based on 21 replicable SVGs identified by STAREG (left: ST Replicate 1 study; right: ST Replicate 8 study). (**b**) The number of replicable SVGs identified by different methods. (**c**) Moran's I statistic of replicable SVGs only identified by STAREG compared to that of not replicable SVGs in the MOB data.

For comparison, we added SPARK-X [Zhu et al., 2021] to analyze the MOB data. After filtering out genes that are expressed in less than 10% of the spatial locations and selecting spatial locations with at least ten total read counts, we obtained 10,373 genes on 265 spots in the Replicate 1 dataset and 9,671 genes on 232 spots in Replicate 8 dataset. We applied SPARK-X separately to the two datasets to obtain two sequences of p-values. Next, we took paired p values for 9,329 genes common in both studies as input for the replicability

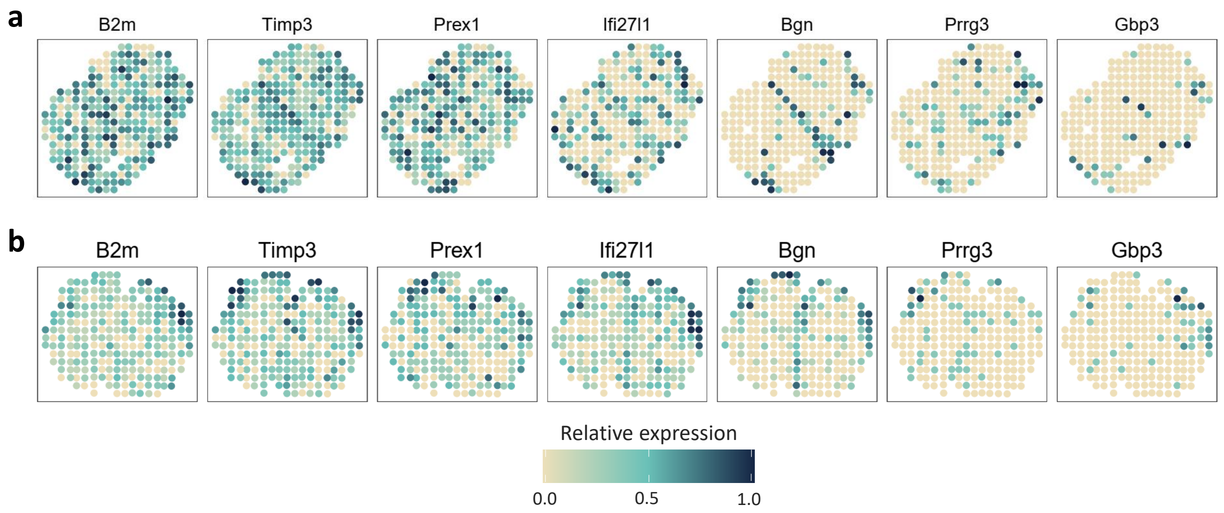

Figure R8: Spatial expression patterns of 7 replicable SVGs uniquely identified by STAREG with SPARK-X based on the (**a**) ST Replicate 1 data and (**b**) ST Replicate 8 data. Different colors represent relative gene expression levels (antique white: low; navy blue: high).

analysis.

Using SPARK-X [Zhu et al., 2021], the results of different methods at the FDR level 0.05 are summarized in Figure R7(b). Overall, by using p-values generated by SPARK-X, all methods were less powerful than those using p-values generated by SPARK. STAREG detected 21 replicable SVGs, 7 of which were not identified by any other methods. All 21 replicable SVGs are identified by STAREG if we use p-values produced by SPARK. MaxP and JUMP identified 2 replicable SVGs, and radjust detected one more. MaRR and *ad hoc* BH identified 7 and 13 replicable SVGs, respectively. We clustered the 21 genes identified by STAREG into three groups with distinct spatial expression patterns using R package *amap* v0.8-18. As shown in Figure R7(a), the three distinct spatial patterns can be matched to three main layers in MOB: Pattern I corresponds to the granular cell layer, Pattern II corresponds to the mitral cell layer and Pattern III corresponds to the glomerular cell layer. We list spatial expression patterns of 7 replicable SVGs uniquely identified by STAREG in Figure R8 as additional evidence. We also calculated the Moran's I statistic [Moran, 1950], a commonly used metric to quantify spatial autocorrelations. As shown in Figure R7(c), the 7 replicable SVGs uniquely identified by STAREG have larger Moran's I statistic than

that of $9,308$ genes not identified by STARGE. We added these in Supplementary Section D.1 on page 18.

5. *In both real data applications, it would be interesting to see more detailed analysis on the replicable SVGs that were only identified by the proposed method. Do they show strong spatial patterns? Do they show consistent patterns in both datasets?*

Response: Thank you for the question. We conducted additional analysis by calculating the Moran's I statistic [Moran, 1950]. Moran's I of the MOB and MC datasets are presented in Figure R9(a) and R9(b), respectively. We observe that the replicable SVGs uniquely identified by STAREG have larger Moran's I than that of genes not detected by STAREG.

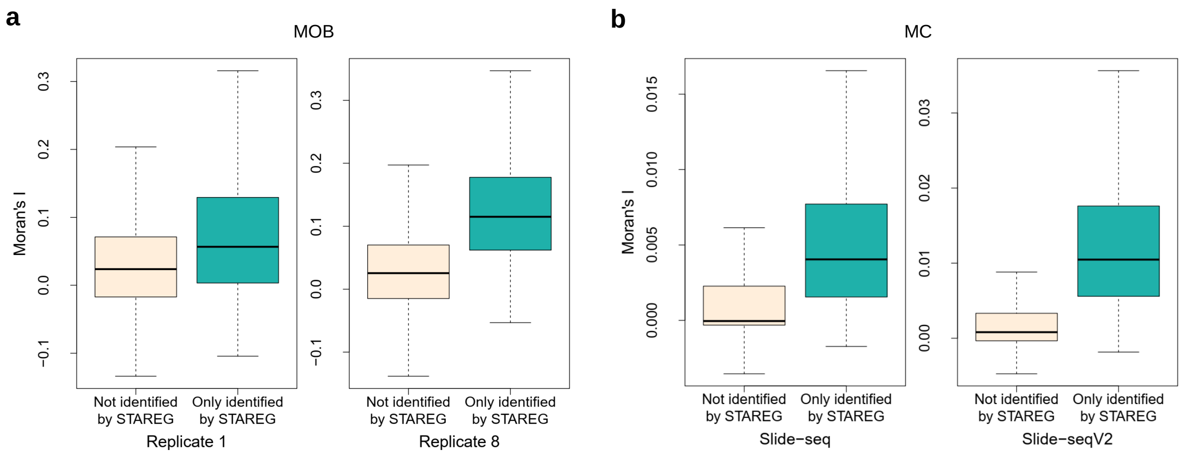

Figure R9: Moran's I statistic of replicable SVGs only identified by STAREG compared to genes not identified by STAREG based on (**a**) ST data from MOB and (**b**) Slide-seq data and Slide-seqV2 data from MC.

We next examined the spatial expression patterns of replicable SVGs uniquely identified by STAREG. Figure R10(a) present three spatial patterns based on 163 replicable SVGs only identified by STAREG in the ST data from MOB (left: Replicate 1 study; right: Replicate 8 study). Spatial expression patterns of 16 randomly selected SVGs in the ST Replicate 1 study and ST Replicate 8 study are listed in Figure R10(b) and R10(c), respectively. The three distinct spatial patterns can be matched to three main layers

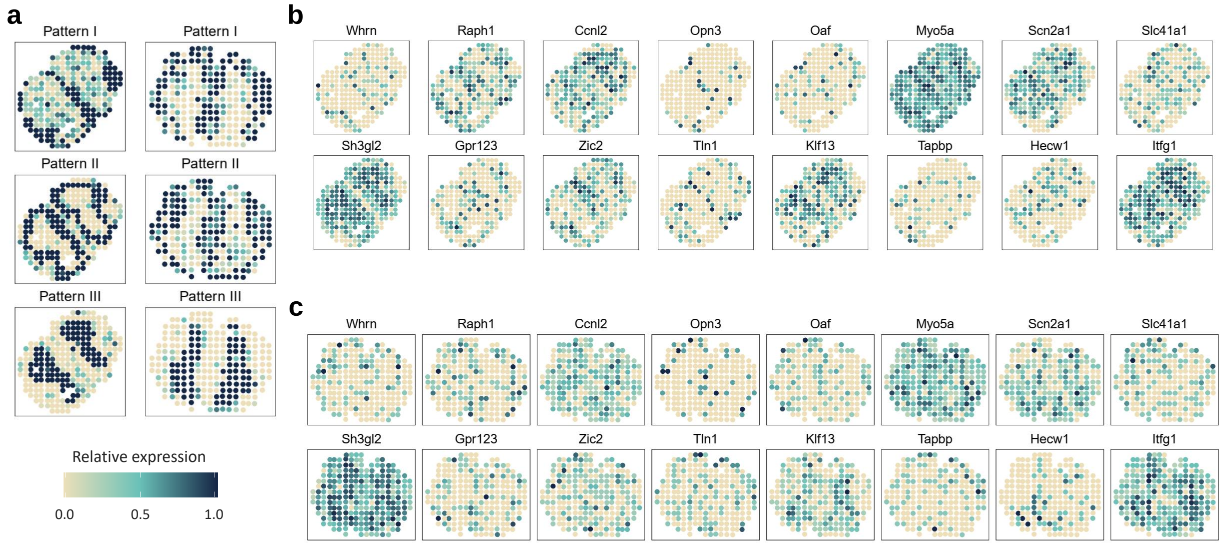

Figure R10: Analysis results of the ST data from MOB. (**a**) Three spatial patterns summarized based on the 163 replicable SVGs uniquely identified by STAREG (left: ST Replicate 1 study; right: ST Replicate 8 study). (**b**) Spatial expression patterns of 16 genes randomly selected from the 163 replicable SVGs uniquely identified by STAREG based on the ST Replicate 1 data. (**c**) Spatial expression patterns of 16 genes randomly selected from the 163 replicable SVGs uniquely identified by STAREG based on the ST Replicate 8 data. Different colors represent relative gene expression levels (antique white: low; navy blue: high).

in MOB: Pattern I corresponds to the granular cell layer, Pattern II corresponds to the mitral cell layer and Pattern III corresponds to the glomerular cell layer. Figure R11(a) presents three spatial patterns summarized based on 341 replicable SVGs only identified by STAREG in the Slide-seq data (left) and Slide-seqV2 data (right) from MC. Spatial expression patterns of 10 randomly selected SVGs in the Slide-seq study and Slide-seqV2 study are listed in Figure R11(b) and R11(c), respectively. We observe spatial patterns in the two studies, with Pattern I corresponding to the spatial distribution of the granular cell layer, Pattern III corresponding to the purkinje cell layer, and Pattern II corresponding to other cell layers.

We added the above results in main Figures 6(a),8(a) and Supplementary Figures S12,S14. Please see the main paper on page 14 lines 227-234 and page 18 lines 301-306.

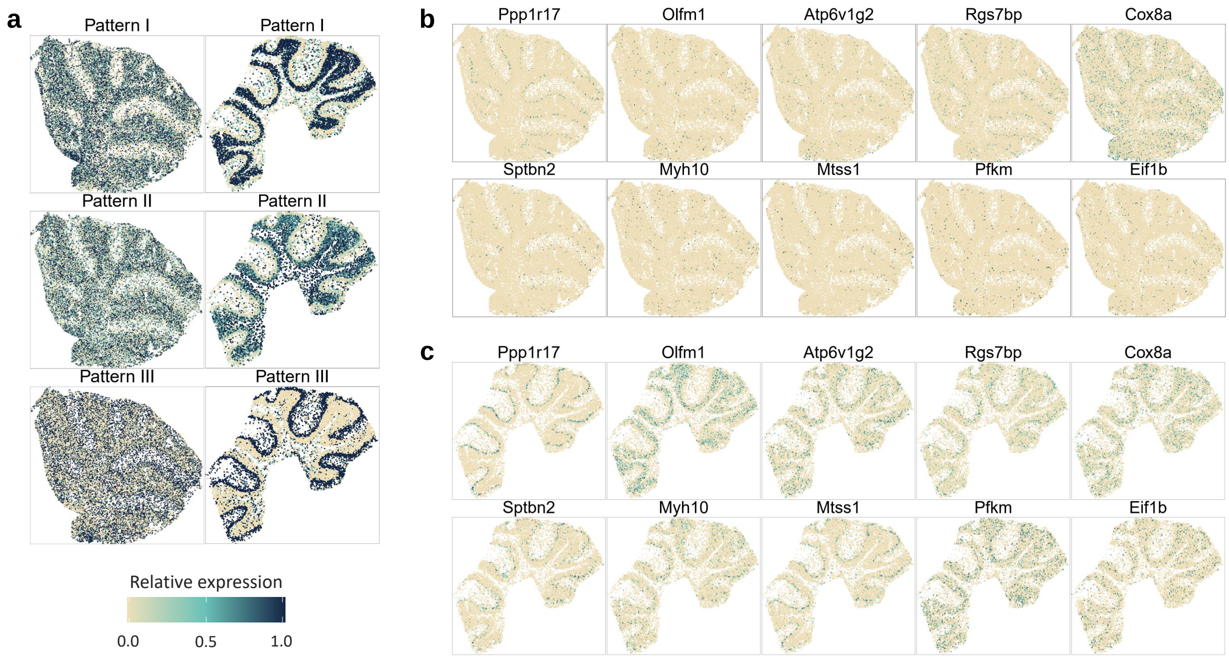

Figure R11: Analysis results of the Slide-seq data and Slide-seqV2 data from MC. (**a**) Three spatial patterns summarized based on the 341 replicable SVGs uniquely identified by STAREG (left: Slide-seq study; right: Slide-seqV2 study). (**b**) Spatial expression patterns of 10 genes randomly selected from the 341 replicable SVGs uniquely identified by STAREG based on the Slide-seq data. (**c**) Spatial expression patterns of 10 genes randomly selected from the 341 replicable SVGs uniquely identified by STAREG based on the Slide-seqV2 data. Different colors represent relative gene expression levels (antique white: low; navy blue: high).

Minor Comments

6. *The authors should provide a more detailed proof or explanation in a supplementary file. For example, for the monotonicity of* $Lfdr(p_1, p_2)$*, the formula below formula (3) (page 19), and the estimation of* f_1 *and* f_2*.*

Response: We have

$$\text{Lfdr}(x_1, x_2) = 1 - \frac{\xi_{11} f_1(x_1) f_2(x_2)}{f(x_1, x_2)},$$

where $f(x_1, x_2) = \xi_{00} f_0(x_1) f_0(x_2) + \xi_{01} f_0(x_1) f_2(x_2) + \xi_{10} f_1(x_1) f_0(x_2) + \xi_{11} f_1(x_1) f_2(x_2)$. If

$x_1 \leq y_1$ and $x_2 \leq y_2$, we aim to show that $\text{Lfdr}(x_1, x_2) \leq \text{Lfdr}(y_1, y_2)$. To this end, we have

$$
\begin{aligned}
& \text{Lfdr}(y_1, y_2) - \text{Lfdr}(x_1, x_2) \\
= & \frac{\xi_{11}\{f_1(x_1)/f_0(x_1)\}\{f_2(x_2)/f_0(x_2)\}}{\xi_{00} + \xi_{01} f_2(x_2)/f_0(x_2) + \xi_{10} f_1(x_1)/f_0(x_1) + \xi_{11}\{f_1(x_1)/f_0(x_1)\}\{f_2(x_2)/f_0(x_0)\}} \\
- & \frac{\xi_{11}\{f_1(y_1)/f_0(y_1)\}\{f_2(y_2)/f_0(y_2)\}}{\xi_{00} + \xi_{01} f_2(y_2)/f_0(y_2) + \xi_{10} f_1(y_1)/f_0(y_1) + \xi_{11}\{f_1(y_1)/f_0(y_1)\}\{f_2(y_2)/f_0(y_0)\}} \\
\geq & \; 0
\end{aligned}
$$

under the assumption that $f_1(x)/f_0(x)$ and $f_2(x)/f_0(x)$ are monotone non-increasing. We next derive the expectation of false discoveries in terms of the expectation of functions of Lfdr. Specifically, we have

$$
\begin{aligned}
& E\left[\sum_{i=1}^{m} I\{\text{Lfdr}_i \leq \lambda\}(1 - \theta_{1i}\theta_{2i})\right] \\
=& \sum_{i=1}^{m} P(\text{Lfdr}_i \leq \lambda, \theta_{1i}\theta_{2i} = 0) \\
=& \sum_{i=1}^{m} \Big\{\xi_{00} P(\text{Lfdr}_i \leq \lambda \mid \theta_{1i} = 0, \theta_{2i} = 0) + \xi_{01} P(\text{Lfdr}_i \leq \lambda \mid \theta_{1i} = 0, \theta_{2i} = 1) \\
& + \xi_{10} P(\text{Lfdr}_i \leq \lambda \mid \theta_{1i} = 1, \theta_{2i} = 0)\Big\} \\
=& \sum_{i=1}^{m} \Big\{\xi_{00} E[I\{\text{Lfdr}_i \leq \lambda \mid \theta_{1i} = 0, \theta_{2i} = 0\}] + \xi_{01} E[I\{\text{Lfdr}_i \leq \lambda \mid \theta_{1i} = 0, \theta_{2i} = 1\}] \\
& + \xi_{10} E[I\{\text{Lfdr}_i \leq \lambda \mid \theta_{1i} = 1, \theta_{2i} = 0\}]\Big\} \\
=& \sum_{i=1}^{m} \Big\{ \int \xi_{00} I\{\text{Lfdr}_i \leq \lambda\} f_0(p_{1i}) f_0(p_{2i}) dp_{1i} dp_{2i} + \int \xi_{01} I\{\text{Lfdr}_i \leq \lambda\} f_0(p_{1i}) f_2(p_{2i}) dp_{1i} dp_{2i} \\
& + \int \xi_{10} I\{\text{Lfdr}_i \leq \lambda\} f_1(p_{1i}) f_0(p_{2i}) dp_{1i} dp_{2i}\Big\} \\
=& \sum_{i=1}^{m} \int [\xi_{00} f_0(p_{1i}) f_0(p_{2i}) + \xi_{01} f_0(p_{1i}) f_2(p_{2i}) + \xi_{10} f_1(p_{1i}) f_0(p_{2i})] I\{\text{Lfdr}_i \leq \lambda\} dp_{1i} dp_{2i} \\
=& \sum_{i=1}^{m} \int f(p_{1i}, p_{2i}) \text{Lfdr}_i I\{\text{Lfdr}_i \leq \lambda\} dp_{1i} dp_{2i} \\
=& E\left[\sum_{i=1}^{m} \text{Lfdr}_i I\{\text{Lfdr}_i \leq \lambda\}\right].
\end{aligned}
$$

Please see the main paper on page 23 lines 374-377 and page 24 for the above derivations. Estimations of f_1 and f_2 are provided in the Supplementary Section A on pages 2-6.

Response to #3 Reviewer's Comments

General Comments. *The authors introduce a novel replicability analysis method for controlling the false discovery rate (FDR) specifically designed for two studies. They highlight that this method is particularly useful when only summary statistics are available, making traditional modeling approaches ineffective. This method can be applied in scenarios where similar studies are conducted or when the same experiment is performed with different modalities within a multi-omics framework. However, they acknowledge a limitation: the method is not suitable for more than two studies, which could impact its usability despite understandable computational constraints.*

1. *The simplicity of the simulation studies in the main manuscript may not fully demonstrate that your method is powerful. I suggest replacing the manuscript's simulation study with the one from the supplementary material D after summarizing the results. In the manuscript's simulation, the p-values are derived from a single Wald-type test statistic. Gene-to-gene correlation, common in various types of omics, requires multiple observations to be accurately reflected, which is not considered in your simulation models. Additionally, in the manuscript model, the rationale for using two parameters, mu and sigma, is unclear since the study essentially uses one parameter (mu/sigma), given that X/sigma = N(mu/sigma, 1). The simulation model in supplementary material D is still not convincing to me because it seems that you are generating DEGs not SVGs, although I understand the challenges in making it realistic. I recommend checking simulation model references such as Wei Vivian Li and Jingyi Jessica Li's "A statistical simulator scDesign for rational scRNA-seq experimental design," Bioinformatics, and Zhu, J., Shang, L., & Zhou, X.'s "SRTsim: spatial pattern preserving simulations for spatially resolved transcriptomics," Genome Biology.*

Response: Thank you for the excellent suggestion. We added a series of realistic simulations by employing the SRTsim simulator [Zhu et al., 2023] developed for spatially resolved transcriptomic data. Please see the response to #2 reviewer's comment #3.

In addition, we simulated data with local dependence to examine the robustness of the STAREG. Specifically, in each study, we divided the $m = 10,000$ genes into 100 blocks and each block was further divided into two sub-blocks of equal size. Within each sub-

block, there is a constant positive correlation (e.g., 0.2) and a constant negative correlation (e.g., −0.2), respectively. Data in different blocks are independent. In each block of study j ($j = 1, 2$), the corresponding hidden states are vector $\boldsymbol{\theta}_j^* = (\theta_{jk}^*)_{k=1}^{100}$, the test statistics were generated from a 100-dimensional multivariate normal distribution: $\boldsymbol{X}_j \sim \text{MVN}(\boldsymbol{\mu}, \Sigma)$, where the mean vector $\boldsymbol{\mu} = \boldsymbol{\theta}_j^* \cdot \mu_j$. We use a 4-dimensional covariance matrix with $\rho = 0.2$ to illustrate the pattern of Σ,

$$\Sigma = \begin{pmatrix} 1 & 0.2 & -0.2 & -0.2 \\ 0.2 & 1 & -0.2 & -0.2 \\ -0.2 & -0.2 & 1 & 0.2 \\ -0.2 & -0.2 & 0.2 & 1 \end{pmatrix}.$$

We set $m = 10,000, \xi_{01} = \xi_{10}$ and $\xi_{11} = 0.05$, and examined the performance of different methods under different μ_1, μ_2, ξ_{00} and ρ. The FDR control and power comparisons at the FDR level 0.05 with different methods are presented in Figure R12. Figure R13 presents the FDR control and power comparison of different methods with varied FDR level when $m = 10,000, \xi_{00} = 0.95, \xi_{11} = 0.02$, $\xi_{01} = \xi_{10} = 0.015, \mu_1 = \mu_2 = 2$ and $\rho = 0.2$. In Figure R12(a), the horizontal dashed lines indicate the target FDR level 0.05. We observe that *ad hoc* BH and MaRR do not control the FDR. Other methods control the FDR across all settings, where MaxP is overly conservative. STAREG has higher power than competing methods.

We added these in Supplementary Section C.1 on pages 14-15.

2. *The authors validated their analysis results by comparing detected genes with a validation gene list in the data analysis. I am curious about how the percentage is calculated in this validation process. I expect it to be calculated as the number of validation genes within each detected genes divided by the total number of validation genes. If so, considering the values in Figures 4-d and 6-d, there may be few or no validation genes among the additionally detected genes. Could you please compare the percentage of validation genes among the additionally detected genes with that of other detected genes? If the additionally detected genes show only minimal overlap with the validation set, can we conclude that the validation process effectively validates the additionally detected genes and guarantees that your method is powerful?*

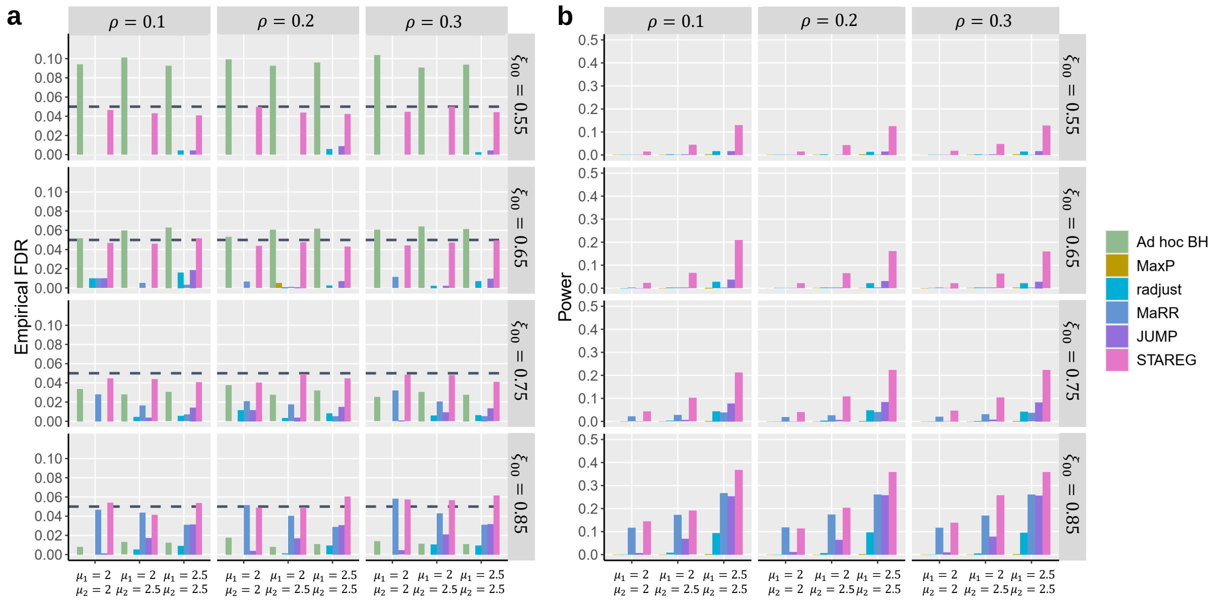

Figure R12: Simulation results of FDR and power for dependent data. Simulations were performed with $m = 10,000, \xi_{11} = 0.05$ and $\xi_{01} = \xi_{10}$ under different μ_1, μ_2, ξ_{00} and ρ. The horizontal dashed line represents the target FDR level of 0.05. We do not present the power of *ad hoc* BH as it fails to control FDR.

Response: Thank you for the question. We revised the validation process to better summarize the results. Specifically, we summarized two lists of reference genes related to MOB from previously published literature to validate replicable SVGs identified by different methods in the MOB data analysis. The validation rate is calculated as the number of validated genes within each detected replicable SVG set divided by the total number of genes in corresponding reference gene list. The first reference gene set from the Allen Brain Atlas [Sunkin et al., 2012] contains 3,485 genes differentially expressed in three main layers in MOB relative to other tissues. As can be seen in the left part of Figure R14(a), STAREG has the highest validation rate (10%), followed by MaRR (8%), JUMP (7%), radjust (7%), *ad hoc* BH (6%) and MaxP (5%). Second, we obtained a list of 2030 cell-type marker genes identified in a recent single-cell RNA sequencing study in MOB [Tepe et al., 2018]. As in the right part of Figure R14(a), STAREG has the highest validation rate of 30% in the Single-cell marker gene list, followed by MaRR (26%), JUMP (24%), radjust (23%), *ad hoc* BH (19%) and MaxP (18%). Furthermore, we examined the

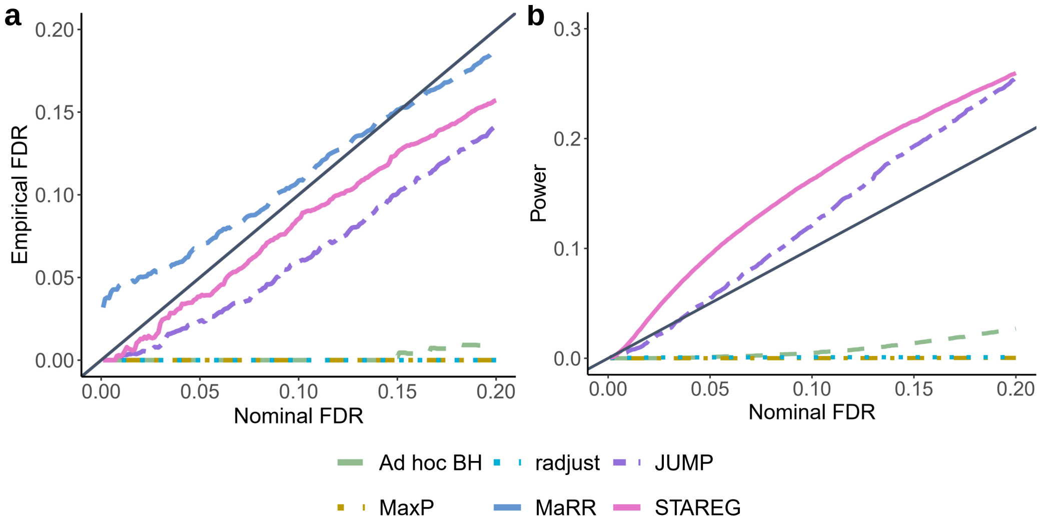

Figure R13: Simulation results of FDR and power for dependent data with varied nominal FDR. Simulaitons were performed with $m = 10,000, \xi_{00} = 0.95, \xi_{11} = 0.02$, $\xi_{01} = \xi_{10} = 0.015, \mu_1 = \mu_2 = 2$ and $\rho = 0.2$.

top-$2,000$ gene set based on the maximum of p-values and the Lfdr estimated by STAREG. We observe that the top-$2,000$ gene set based on Lfdr has higher validation rate in both reference gene lists (Figure R14(b)).

We next validated the quality of replicable SVGs identified by different methods in the MC data analysis using two lists of MC-related genes. The first validation set consists of $2,431$ genes related to MC from the BioGPS mouse cell type and tissue gene expression profiles dataset [Wu et al., 2013]. As in the left part of Figure R14(c), 10% of genes detected by STAREG were validated. The validation rates of the other methods, in descending order, were 6% (*ad hoc* BH and adjust), 5% (JUMP), and 4% (MaxP and MaRR). Second, we obtained a list of differentially expressed genes across all cell clusters in mouse cerebellar cortex from Kozareva et al. [2021]. Let $\log \mathrm{FC}$ denote logarithmic fold changes. By filtering out genes with $|\log \mathrm{FC}| < 1.5$, we used a final set of 995 marker genes that are differentially expressed across all cell clusters in mouse cerebellar cortex for the validation. Figure R14(c) (right) shows the validation results. STAREG has the highest validation rate of 20%, followed by *ad hoc* BH (12%), radjust (12%), JUMP (11%), MaRR (11%) and MaxP (11%). We also checked the top-$2,000$ gene set based on the maximum of p-values and the Lfdr estimated by STAREG. As in Figure R14(d), the top-$2,000$ gene set based on Lfdr

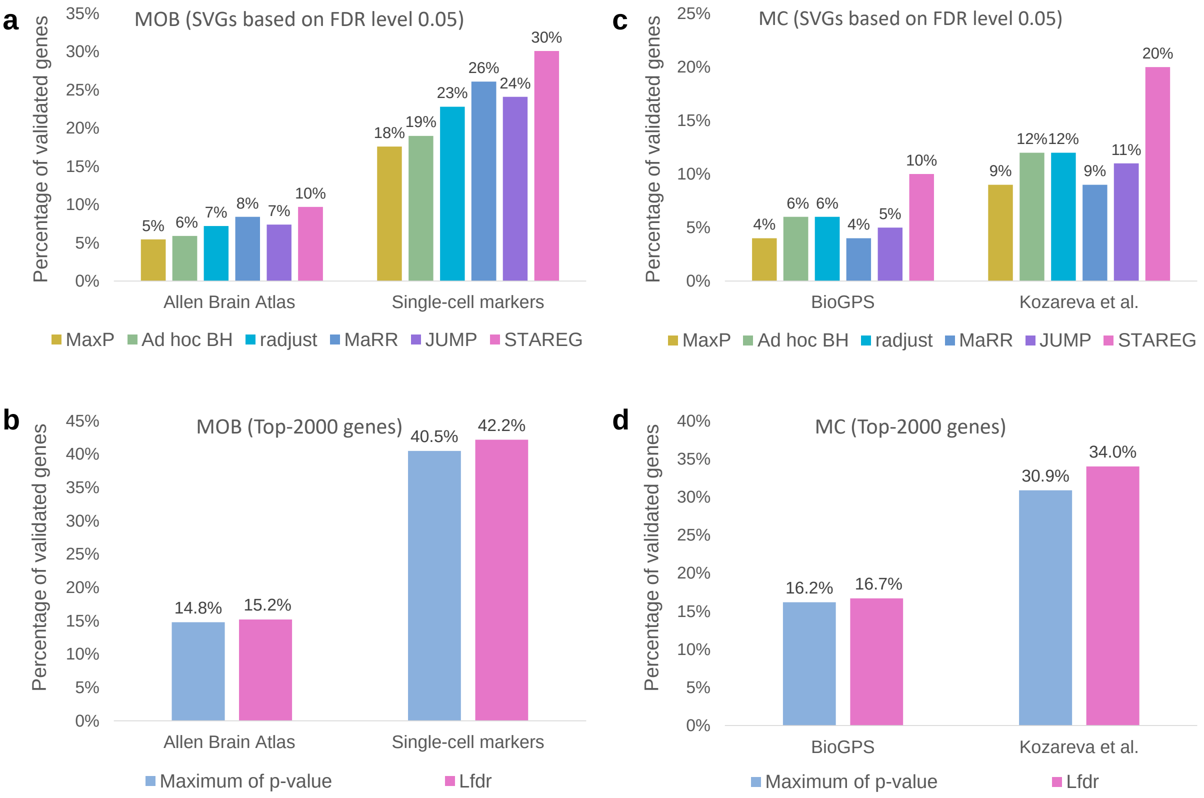

Figure R14: Validation results based on replicable SVGs identified by different methods in previously published reference gene lists. (**a**) The percentage of validated genes in the corresponding reference gene list based on replicable SVGs identified by different methods in the MOB data analysis. (**b**) The percentage of validated genes based on top-2,000 gene set calculated from the maximum of p-values and Lfdr in the MOB data analysis. (**c**) The percentage of validated genes in the corresponding reference gene list based on replicable SVGs identified by different methods in the MC data analysis. (**d**) The percentage of validated genes based on top-2,000 gene set calculated from the maximum of p-values and Lfdr in the MC data analysis.

has higher validation rate in both reference gene lists.

We added the above results in Figures 6(b),8(b) and Supplementary Figures 13(a),15(a). Please see the main paper pages 14-15 lines 235-249 and pages 18-20 lines 307-321.

3. *I prefer to conduct gene enrichment tests using all the sorted genes because it is*

not sensitive to the cutoff values. If it is possible with the proposed method, comparing the number of significant GO terms using sorted adjusted p-values would be valuable. Additionally, incorporating AUC values into the simulation study would further justify our approach.

Response: Thank you for the suggestions. We performed gene set enrichment analysis (GSEA) for the gene ontology (GO) gene sets [Aleksander et al., 2023] with the R package *fgsea* [Korotkevich et al., 2021]. STAREG ranked genes based on the average of the order statistics of Lfdr. Specifically, let $\widehat{\text{Lfdr}}_{(1)} \leq \ldots \leq \widehat{\text{Lfdr}}_{(m)}$ be the order statistics of $\{\widehat{\text{Lfdr}}_i\}_{i=1}^m$, then genes were ranked based on $\frac{1}{i}\sum_{j=1}^{i} \widehat{\text{Lfdr}}_{(j)}, i = 1, \ldots, m$. In comparison, a common strategy for ranking genes is using p-values. Specifically, suppose p_i is the maximum of p-values from the two studies for the ith gene, and $p_{(1)} \leq \ldots \leq p_{(m)}$ denote the order statistics. Genes were ranked based on $mp_{(i)}/i, i = 1, \ldots, m$.

In the GO GSEA of MOB data, using Lfdr based ranking, 781 GO terms were enriched at FDR cutoff 0.05 (Figure R15), including 4 of the 5 GO terms identified by p-value based ranking. Many of the additional identified GO terms are related to synapse assembly, dendrite morphogenesis, neuron differentiation, and regulation of G-protein, which play critical roles in olfactory bulb organization and olfactory signal transduction [Ebrahimi and Chess, 1998].

In the GO GSEA of MC data, at FDR cutoff 0.05, 448 GO terms were enriched by using Lfdr based ranking (Figure R16), while 6 GO terms were identified by p-value based ranking. The GO terms enriched by adjusted Lfdr reveal critical roles of synaptic signaling, axon development, neuron projection, and cerebellar cortex development for the cerebellum structure and functions.

We didn't show area under curve (AUC) values in the simulation studies as the horizontal axis in the plots is empirical FDR, which is different from the receiver operator characteristic (ROC) curve (with false positive rate as the horizontal axis). We performed additional simulations to plot ROC curves under different settings with corresponding AUC values (Figure R2(b) and Figure R17).

We added these results in main Figure 6(c)(d) and Figure 8(c)(d). Please see the main paper pages 15-16 lines 250-262 and page 20 lines 322-329.

4. *The primary limitation is that the current version of the method can only analyze two*

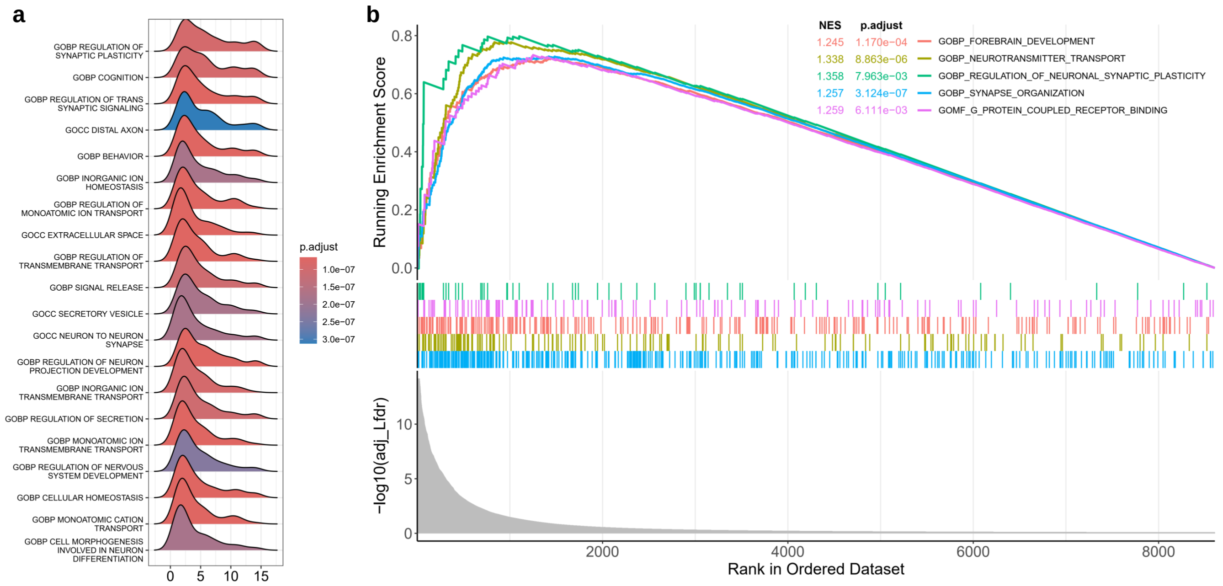

Figure R15: GO GSEA results of the MOB data using $-\log_{10}(\frac{1}{i}\sum_{j=1}^{i}\widehat{\text{Lfdr}}_{(j)})$ as the ranking metric. (**a**) The ridge plot shows the distribution of genes in the top-20 gene sets enriched by STAREG. (**b**) The running enrichment scores of five representative gene sets only enriched by STAREG at FDR cutoff 0.05.

studies due to time constraints. However, it seems that if you redefine replicability to require genes to be identified as markers in at least two studies across multiple studies, it could potentially reduce computational time, as illustrated by the definition of replicability analysis in Hoang, A. T., & Dickhaus, T. (2022). Randomized-values for multiple testing and their application in replicability analysis. Biometrical Journal, 64(2), 384-409: "replicability analysis, where one aims at identifying discoveries made across more than one of $s > 2$ *given independent studies". This could make the method more broadly applicable. I would appreciate your thoughts on this. Additionally, please consider incorporating relevant references from the RNA-seq literature within this paper.*

Response: The strength of STAREG is mainly attributed to the fact that it enumerates all possible joint hidden states and considers heterogeneity across studies. Even if we consider the partial conjunction null (i.e., identifying discoveries made across more than r of $n \geq 2$ studies), it still requires the estimation of all joint state proportions and non-null p-value distributions. For instance, if the number of studies $n = 3$ and $r = 2$, let

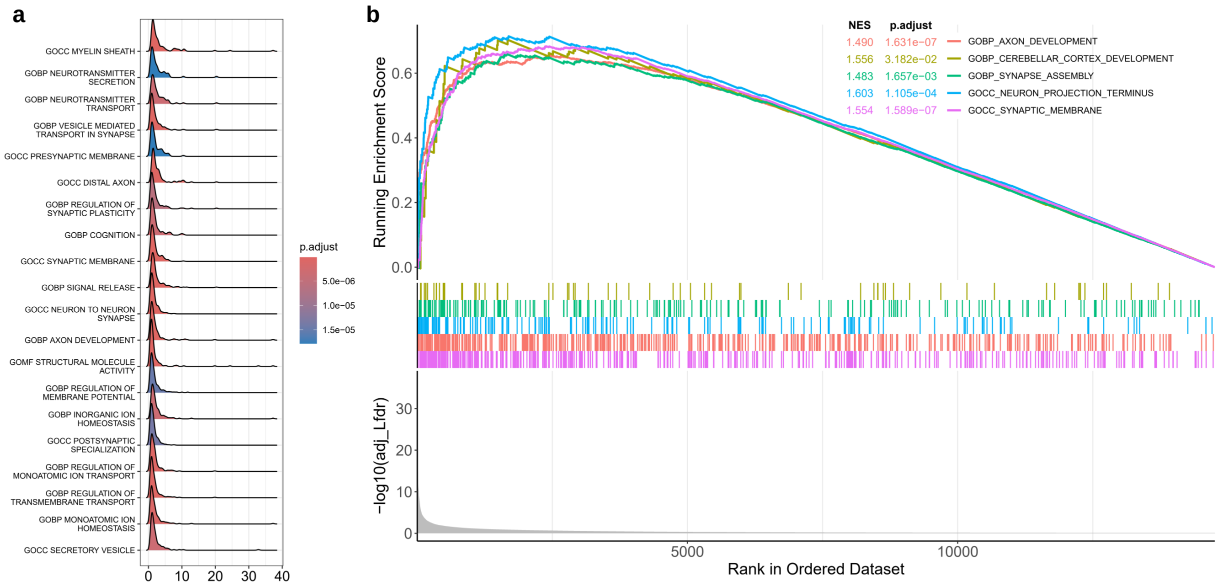

Figure R16: GO GSEA results of the MC data using $-\log_{10}(\frac{1}{i}\sum_{j=1}^{i}\widehat{\text{Lfdr}}_{(j)})$ as the ranking metric. (**a**) The ridge plot shows the distribution of genes in the top-20 gene sets enriched by STAREG. (**b**) The running enrichment scores of five representative gene sets only enriched by STAREG at FDR cutoff 0.05.

$f = \xi_{000}f_0f_0f_0 + \xi_{001}f_0f_0f_3 + \xi_{010}f_0f_2f_0 + \xi_{011}f_0f_2f_3 + \xi_{100}f_1f_0f_0 + \xi_{101}f_1f_0f_3 + \xi_{110}f_1f_2f_0 + \xi_{111}f_1f_2f_3$. The Lfdr for our replicability null hypothesis is defined as

$$\text{Lfdr}_i(p_{1i}, p_{2i}, p_{3i}) = 1 - \frac{\xi_{111}f_1f_2f_3}{f},$$

and the Lfdr for the partial conjunction test is written as

$$\text{Lfdr}_i(p_{1i}, p_{2i}, p_{3i}) = \frac{\xi_{000}f_0f_0f_0 + \xi_{001}f_0f_0f_3 + \xi_{010}f_0f_2f_0 + \xi_{100}f_1f_0f_0}{f}.$$

Therefore, using the partial conjunction test under our framework does not reduce the computational cost. We extended STAREG to the general case of n studies ($n \geq 2$) and explored the computational time required for the replicability analysis of 10,000 genes. STAREG is feasible for 10 studies or so. Please see the response to #2 reviewer's comment #2. Our main application is on spatial transcriptomic data, not RNA-seq data. Despite this, we added one single-cell data analysis and related literature can be found there.

Minor Comments

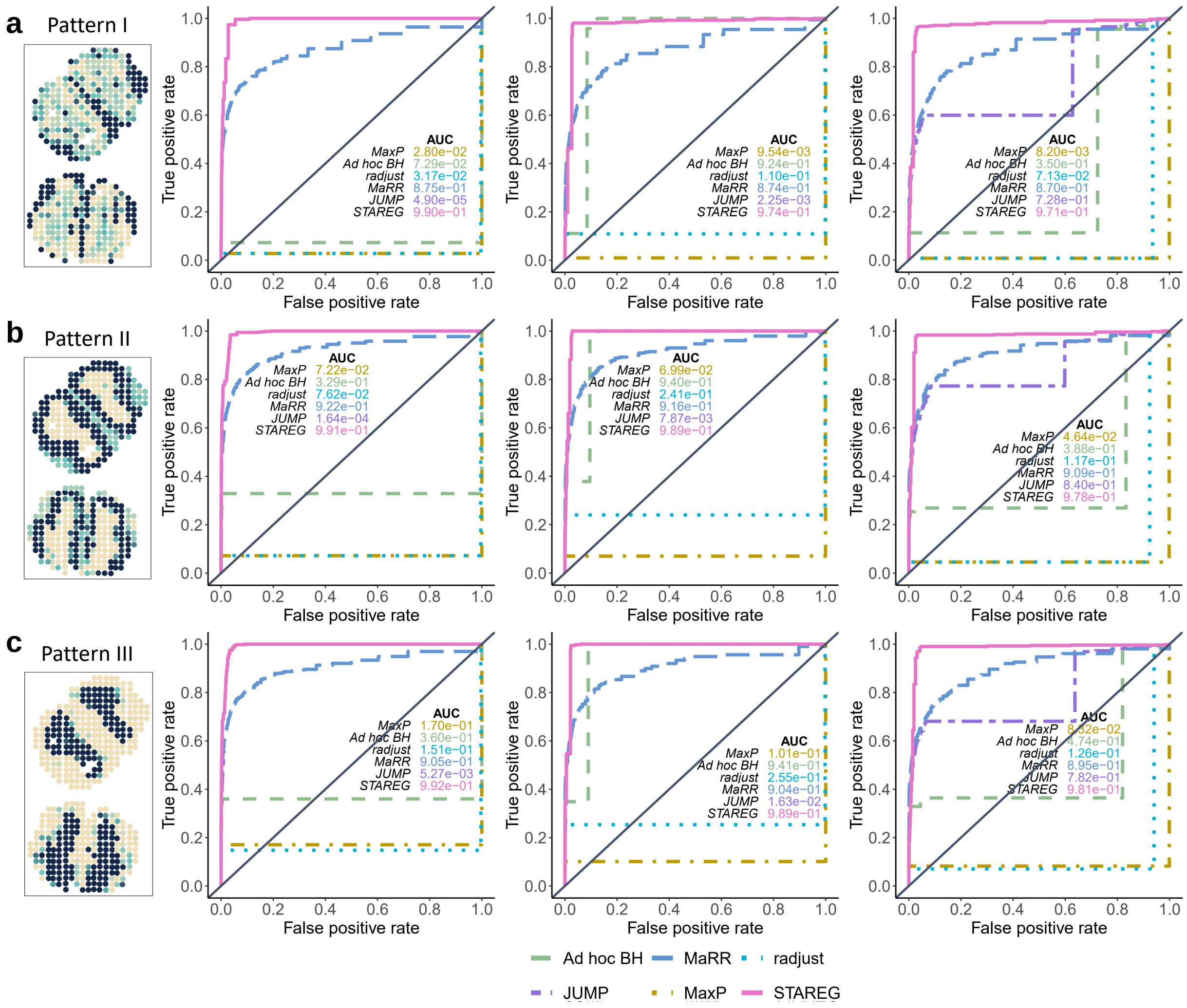

Figure R17: ROC curves and AUC values in the realistic simulation studies based on SRT data [Ståhl et al., 2016]. Simulations were performed under the setting of $m = 10,000, \xi_{00} = 0.9, \xi_{01} = \xi_{10} = 0.025$ and $\xi_{11} = 0.05$. The signal strengths were set to be strong for study 1 and weak for study 2 across all simulations. In each setting, two SRT datasets were independently generated from corresponding MOB replicates with different noise levels (top: $\tau_1 = \tau_2 = 0.2$; middle: $\tau_1 = 0.2, \tau_2 = 0.5$; bottom: $\tau_1 = \tau_2 = 0.5$). Each row corresponds to a different spatial expression pattern. (**a**) Pattern I; (**b**) Pattern II; (**c**) Pattern III. The diagonal line with slope 1 is used as a reference.

5. *Include more explanation on the figure in the caption of Figure 1 and Figure S1.*

Response: Done.

References

S. A. Aleksander, J. Balhoff, S. Carbon, J. M. Cherry, H. J. Drabkin, D. Ebert, M. Feuermann, P. Gaudet, N. L. Harris, D. P. Hill, and et al. The gene ontology knowledgebase in 2023. *Genetics*, 224(1):iyad031, 03 2023. ISSN 1943-2631. doi: 10.1093/genetics/iyad031. URL `https://doi.org/10.1093/genetics/iyad031`.

S. Das, A. Rai, M. L. Merchant, M. C. Cave, and S. N. Rai. A comprehensive survey of statistical approaches for differential expression analysis in single-cell rna sequencing studies. *Genes*, 12(12):1947, 2021.

F. A. Ebrahimi and A. Chess. Olfactory G proteins: Simple and complex signal transduction. *Current Biology*, 8(12):R431–R433, 1998.

O. Franzén, L.-M. Gan, and J. L. M. Björkegren. PanglaoDB: a web server for exploration of mouse and human single-cell rna sequencing data. *Database*, 2019:baz046, 2019.

G. Korotkevich, V. Sukhov, N. Budin, B. Shpak, M. N. Artyomov, and A. Sergushichev. Fast gene set enrichment analysis. *bioRxiv*, 2021. doi: 10.1101/060012. URL `https://www.biorxiv.org/content/early/2021/02/01/060012`.

V. Kozareva, C. Martin, T. Osorno, S. Rudolph, C. Guo, C. Vanderburg, N. Nadaf, A. Regev, W. G. Regehr, and E. Macosko. A transcriptomic atlas of mouse cerebellar cortex comprehensively defines cell types. *Nature*, 598(7879):214–219, 2021.

P. A. Moran. Notes on continuous stochastic phenomena. *Biometrika*, 37(1/2):17–23, 1950.

P. L. Ståhl, F. Salmén, S. Vickovic, A. Lundmark, J. F. Navarro, J. Magnusson, S. Giacomello, M. Asp, J. O. Westholm, M. Huss, et al. Visualization and analysis of gene expression in tissue sections by spatial transcriptomics. *Science*, 353(6294):78–82, 2016.

S. Sun, J. Zhu, and X. Zhou. Statistical analysis of spatial expression patterns for spatially resolved transcriptomic studies. *Nature Methods*, 17(2):193–200, 2020.

S. M. Sunkin, L. Ng, C. Lau, T. Dolbeare, T. L. Gilbert, C. L. Thompson, M. Hawrylycz, and C. Dang. Allen Brain Atlas: an integrated spatio-temporal portal for exploring the central nervous system. *Nucleic Acids Research*, 41(D1):D996–D1008, 2012.

B. Tepe, M. C. Hill, B. Pekarek, P. J. Hunt, T. J. Martin, J. F. Martin, and B. R. Arenkiel. Single-cell RNA-seq of mouse olfactory bulb reveals cellular heterogeneity and activity-dependent molecular census of adult-born neurons. *Cell Reports*, 25(10):2689–2703, 2018.

C. Wu, I. MacLeod, and A. I. Su. BioGPS and MyGene.info: organizing online, gene-centric information. *Nucleic Acids Research*, 41(D1):D561–D565, 2013.

J. Zhu, S. Sun, and X. Zhou. SPARK-X: non-parametric modeling enables scalable and robust detection of spatial expression patterns for large spatial transcriptomic studies. *Genome Biology*, 22(1):1–25, 2021.

J. Zhu, L. Shang, and X. Zhou. SRTsim: spatial pattern preserving simulations for spatially resolved transcriptomics. *Genome Biology*, 24(1):39, 2023.
