# Supplementary material for: STAREG: Statistical replicability analysis of high throughput experiments with applications to spatial transcriptomic studies
Source: PLoS Genet. 2024 Oct 3;20(10):e1011423. doi: 10.1371/journal.pgen.1011423 (PMC11478871; doi:10.1371/journal.pgen.1011423)
Supplement: Attachment — Submitted filename: R2_Response letter.pdf [file pgen.1011423.s007.pdf]

Dear Editor,

A revised version of our paper (PGENETICS-D-24-00265R1) and our response to the referee's comments are attached. We want to thank you and the referees for the very detailed and helpful comments that led to an improved revision.

Yours sincerely,

Li, Y., Zhou, X., Chen, Rui, Zhang, X. and Cao, H.

Response to #2 Reviewer's Comments

Comments. *I appreciate the authors' response to my previous comments and they have addressed most of my concerns. I just have one additional question. The revised manuscript presents a new analysis to identify marker genes from two mouse aortic leukocyte samples. The analysis reveals that STAREG identified 162 genes that were not detected by any other method in the comparison. Can the authors demonstrate these 162 genes were indeed more highly expressed in T memory cells and biologically relevant?*

Response: First, we used a publicly available database, PanglaoDB [Franzén et al., 2019] (`https://panglaodb.se/samples.html`), to validate the results. Among the 162 replicable markers uniquely identified by STAREG, 30 were reported by PanlaoDB as differentially expressed in T memory cells in mouse aortic leukocytes (Table R1). Second, we used papers in the literature to validate the remaining 132 markers, many of which have been demonstrated to be related to T memory cell functions. For instance, *Alcam* regulates multiple effector memory T cell functions via the *Cd6-Alcam* signaling pathway [Ampudia et al., 2020]; *Cd79a* is involved in a composite costimulatory domain *Cd79a/Cd40*, which enriches genes correlated with T-cell proliferation, interferon signaling pathway, naïve and memory T-cell signatures, and T-cell exhaustion [Ung et al., 2022]; *Trdv2-2* is a T cell receptor that can regulate T memory cells, and it influences the multiple fates of T cells in the memory pool by interacting with peptide/*MHC* [Corse et al., 2011, Daniels and Teixeiro, 2015].

Table R1: The 30 validated replicable cell markers uniquely identified by STAREG.

1	*Adgre5*	7	*Ctsb*	13	*Gnb2l1*	19	*Map3k1*	25	*Rpsaps10*
2	*Arl6ip5*	8	*Ddx5*	14	*Hnrnpab*	20	*Ppp1r11*	26	*Selplg*
3	*Bsg*	9	*Efhd2*	15	*Ict1*	21	*Rpl3*	27	*Sf3b2*
4	*Capzb*	10	*Fam49b*	16	*Il21r*	22	*Rpl36aps2*	28	*Smad7*
5	*Cited2*	11	*Fyb*	17	*Kif5b*	23	*Rps17*	29	*Tax1bp1*
6	*Clic1*	12	*Gm9493*	18	*Lsp1*	24	*Rpsa*	30	*Tpt1*

Please see Supplementary Section D.2 pages 19-20 lines 177-188.

References

J. Ampudia, W. W. Young-Greenwald, J. Badrani, S. Gatto, A. Pavlicek, T. Doherty, S. Connelly, and C. T. Ng. CD6-ALCAM signaling regulates multiple effector/memory T cell functions. *The Journal of Immunology*, 204(1_Supplement):150–13, 2020.

E. Corse, R. A. Gottschalk, and J. P. Allison. Strength of TCR–peptide/MHC interactions and in vivo T cell responses. *The Journal of Immunology*, 186(9):5039–5045, 2011.

M. A. Daniels and E. Teixeiro. TCR signaling in T cell memory. *Frontiers in Immunology*, 6:617, 2015.

O. Franzén, L.-M. Gan, and J. L. M. Björkegren. PanglaoDB: a web server for exploration of mouse and human single-cell rna sequencing data. *Database*, 2019:baz046, 2019.

S. Ung, P. Choochuen, W. Khopanlert, K. Maneechai, S. Sangkhathat, S. Terakura, and J. Julamanee. Enrichment of T-cell proliferation and memory gene signatures of CD79A/CD40 costimulatory domain potentiates CD19CAR-T cell functions. *Frontiers in Immunology*, 13, 2022.
