# Supplementary material for: STAREG: Statistical replicability analysis of high throughput experiments with applications to spatial transcriptomic studies
Source: PLoS Genet. 2024 Oct 3;20(10):e1011423. doi: 10.1371/journal.pgen.1011423 (PMC11478871; doi:10.1371/journal.pgen.1011423)
Supplement: Attachment — Submitted filename: Review.docx [file pgen.1011423.s005.docx]

The authors introduce a novel replicability analysis method for controlling the false discovery rate (FDR) specifically designed for two studies. They highlight that this method is particularly useful when only summary statistics are available, making traditional modeling approaches ineffective. This method can be applied in scenarios where similar studies are conducted or when the same experiment is performed with different modalities within a multi-omics framework. However, they acknowledge a limitation: the method is not suitable for more than two studies, which could impact its usability despite understandable computational constraints.

1. The simplicity of the simulation studies in the main manuscript may not fully demonstrate that your method is powerful. I suggest replacing the manuscript’s simulation study with the one from the supplementary material D after summarizing the results. In the manuscript’s simulation, the p-values are derived from a single Wald-type test statistic. Gene-to-gene correlation, common in various types of omics, requires multiple observations to be accurately reflected, which is not considered in your simulation models. Additionally, in the manuscript model, the rationale for using two parameters, mu and sigma, is unclear since the study essentially uses one parameter (mu/sigma), given that X/sigma = N(mu/sigma, 1). The simulation model in supplementary material D is still not convincing to me because it seems that you are generating DEGs not SVGs, although I understand the challenges in making it realistic. I recommend checking simulation model references such as Wei Vivian Li and Jingyi Jessica Li's "A statistical simulator scDesign for rational scRNA-seq experimental design," Bioinformatics, and Zhu, J., Shang, L., & Zhou, X.'s "SRTsim: spatial pattern preserving simulations for spatially resolved transcriptomics," Genome Biology.
2. The authors validated their analysis results by comparing detected genes with a validation gene list in the data analysis. I am curious about how the percentage is calculated in this validation process. I expect it to be calculated as the number of validation genes within each detected genes divided by the total number of validation genes. If so, considering the values in Figures 4-d and 6-d, there may be few or no validation genes among the additionally detected genes. Could you please compare the percentage of validation genes among the additionally detected genes with that of other detected genes? If the additionally detected genes show only minimal overlap with the validation set, can we conclude that the validation process effectively validates the additionally detected genes and guarantee that your method is powerful?
3. I prefer to conduct gene enrichment tests using all the sorted genes because it is not sensitive to the cutoff values. If it is possible with the proposed method, comparing the number of significant GO terms using sorted adjusted p-values would be valuable. Additionally, incorporating AUC values into the simulation study would further justify our approach.
4. The primary limitation is that the current version of the method can only analyze two studies due to time constraints. However, it seems that if you redefine replicability to require genes to be identified as markers in at least two studies across multiple studies, it could potentially reduce computational time, as illustrated by the definition of replicability analysis in Hoang, A. T., & Dickhaus, T. (2022). Randomized‐values for multiple testing and their application in replicability analysis. *Biometrical Journal*, *64*(2), 384-409: “replicability analysis, where one aims at identifying discoveries made across more than one of s>2 given independent studies”. This could make the method more broadly applicable. I would appreciate your thoughts on this. Additionally, please consider incorporating relevant references from the RNA-seq literature within this paper.

Minor Comments

1. Include more explanation on the figure in the caption of Figure 1 and Figure S1.
